# Hepatic transcriptome implications for palm fruit juice deterrence of type 2 diabetes mellitus in young male Nile rats

**DOI:** 10.1186/s12263-016-0545-z

**Published:** 2016-10-22

**Authors:** Soon-Sen Leow, Julia Bolsinger, Andrzej Pronczuk, K. C. Hayes, Ravigadevi Sambanthamurthi

**Affiliations:** 1 Malaysian Palm Oil Board, No. 6, Persiaran Institusi, Bandar Baru Bangi, 43000 Kajang, Selangor Malaysia; 2 Brandeis University, 415 South Street, Waltham, MA 02454 USA

**Keywords:** Palm fruit juice, Oil palm phenolics, Antioxidants, Diabetes, Metabolic syndrome, Gene expression, Nile rat

## Abstract

**Background:**

The Nile rat (NR, *Arvicanthis niloticus*) is a model of carbohydrate-induced type 2 diabetes mellitus (T2DM) and the metabolic syndrome. A previous study found that palm fruit juice (PFJ) delayed or prevented diabetes and in some cases even reversed its early stages in young NRs. However, the molecular mechanisms by which PFJ exerts these anti-diabetic effects are unknown. In this study, the transcriptomic effects of PFJ were studied in young male NRs, using microarray gene expression analysis.

**Methods:**

Three-week-old weanling NRs were fed either a high-carbohydrate diet (%En from carbohydrate/fat/protein = 70:10:20, 16.7 kJ/g; *n* = 8) or the same high-carbohydrate diet supplemented with PFJ (415 ml of 13,000-ppm gallic acid equivalent (GAE) for a final concentration of 5.4 g GAE per kg diet or 2.7 g per 2000 kcal; *n* = 8). Livers were obtained from these NRs for microarray gene expression analysis using Illumina MouseRef-8 Version 2 Expression BeadChips. Microarray data were analysed along with the physiological parameters of diabetes.

**Results:**

Compared to the control group, 71 genes were up-regulated while 108 were down-regulated in the group supplemented with PFJ. Among hepatic genes up-regulated were apolipoproteins related to high-density lipoproteins (HDL) and genes involved in hepatic detoxification, while those down-regulated were related to insulin signalling and fibrosis.

**Conclusion:**

The results obtained suggest that the anti-diabetic effects of PFJ may be due to mechanisms other than an increase in insulin secretion.

## Background

Nutritional overload and sedentary lifestyle give rise to the prevalence of type 2 diabetes mellitus (T2DM) in modern societies, and this chronic disease is estimated to reach 439 million cases by 2030 [87]. Although T2DM is a disease of adults, it is an increasingly common diagnosis among adolescents in high-risk countries such as Asia, the Middle East, and the USA [46]. T2DM is characterised by insulin resistance, declining insulin production and eventual pancreatic β cell failure [71]. This leads to a decrease in glucose transport into liver, muscle and fat cells and an increase in circulating glucose. T2DM is often associated with increasing obesity, via a combination of clinical abnormalities known as the metabolic syndrome, which comprises insulin resistance, visceral adiposity, hypertension, atherogenic dyslipaemia and endothelial dysfunction [32]. These conditions are interrelated and share common mediators, pathways and pathophysiological mechanisms [50]. The metabolic syndrome is a state of chronic low-grade inflammation linked to aberrant energy metabolism as a consequence of complex interplay between genetic and environmental factors [57].

Due to the growing concern over T2DM and the metabolic syndrome, animal models that mimic these human diseases are needed to assess possible anti-diabetic preventative or therapeutic measures [128]. The Nile rat (NR), also known as the African grass rat (*Arvicanthis niloticus*), has been described as a relevant model of T2DM and the metabolic syndrome, as it allows for detailed nutritional modelling of diet-induced T2DM similar to that in humans. The NR spontaneously develops hyperinsulinaemia, hyperglycaemia with dyslipaemia and hypertension in the early phase of the disease [14, 16, 21, 85]. Further characterisation revealed that NRs develop liver steatosis, abdominal fat accumulation, nephropathy, atrophy of pancreatic islets of Langerhans and fatty streaks in the aorta, as well as hypertension [14, 16, 21, 85]. Males are more prone than females, with rapid progression to T2DM depending on the glycaemic load of the challenge diet and cumulative glycaemic load [15]. Although diet challenge appears as the primary factor and dietary intervention can modulate the development of T2DM and metabolic syndrome in NRs, genetic susceptibility also plays a pivotal role, similar to humans. This rodent model thus represents a novel system of gene-diet interactions affecting energy utilisation that can provide insights into the prevention and treatment of diabetes, as well as the metabolic syndrome [14, 21]. As in humans, the NR is sensitive to the daily glycaemic load and as such reliably mirrors the course of T2DM and the metabolic syndrome observed in humans [14].

At present, no cure has been found for T2DM and the metabolic syndrome. Treatment methods normally suggested include lifestyle modifications, treatment of obesity that induces weight reduction, oral anti-diabetic medication that reduces intestinal glucose absorption, increases insulin sensitivity or exerts insulin-sensitising effects or lastly insulin injections [87]. All the above measures have been shown to prevent T2DM in the NR. However, current research strongly supports the concept that the consumption of certain fruits and plant-derived foods is inversely correlated with prevalence of T2DM and the metabolic syndrome [8, 35, 80]. A great array of phenolic compounds may exert anti-diabetic effects either directly or indirectly [1]. Phenolic compounds may influence glucose metabolism by several mechanisms, such as inhibition of carbohydrate digestion and glucose absorption in the small intestine, stimulation of insulin secretion from pancreatic β cells, modulation of hepatic gluconeogenesis, activation of insulin receptors and glucose uptake in insulin-sensitive tissues (thus enhancing insulin sensitivity) and modulation of gut flora activity, as well as modulation of intracellular signalling pathways and gene expression influencing glucose utilisation [26, 47, 79]. Some examples of plant phenolic compounds which were found to display anti-diabetic effects in humans include resveratrol [82, 110], olive leaf extracts [28, 125], pomegranate juice [88] and green tea extracts [61, 69].

The oil palm (*Elaeis guineensis*) fruit contains phenolic compounds [99], which are extracted from the aqueous vegetation liquor produced during oil palm milling. Palm fruit juice (PFJ) consists mainly of phenolic acids, including three caffeoylshikimic acid isomers and *p*-hydroxybenzoic acid [99]. PFJ has been shown to display antioxidant properties and confer positive outcomes on degenerative diseases in various animal models without evidence of toxicity [16, 22, 65–68, 99, 100, 103]. In relation to T2DM, we have previously shown that PFJ blocked T2DM progression in 12-week-old male NRs, with a substantial decrease in blood glucose after 17 weeks of treatment [100]. In addition, PFJ delayed T2DM onset or completely prevented it during the intervention period and even reversed advancing T2DM in young NRs [16]. PFJ has also been shown to deter T2DM complications, including retinopathy and nephropathy in NRs [14, 21, 85], not unlike other plant polyphenols [5]. PFJ thus has demonstrated anti-diabetic effects. However, the detailed molecular mechanisms by which PFJ effects these changes in NRs have yet to be explored, prompting the microarray gene expression analysis in the present study.

## Methods

### Animal feeding and sample collection

Three-week-old male NRs (*n = 16*) were divided into two groups, controls without PFJ (*n = 8*) and PFJ (*n = 8*). We chose to study 3-week-old Nile rats for 4 weeks in this study as this window of development is the most sensitive to the development of nutritionally induced T2DM in the NR and thus provides the highest chances of altering this development through the application of PFJ. This would help pinpoint the gene expression changes caused by PFJ in deterring the occurrence of diabetes more efficiently [16, 21]. Early diabetes (7 weeks of age) in Nile rats is detected by random blood glucose levels, whereas diabetic fasting blood glucose does not always manifest until 12 weeks of age [85]. In addition, only males were used in this experiment as they develop T2DM more readily than females, presumably based on sex hormone differences [21]. NRs in the control group were fed a semi-purified high-carbohydrate diet ad libitum (% En from carbohydrate/fat/protein = 70:10:20, 16.7 kJ/g), while those in the PFJ group were given liquid PFJ incorporated directly into the same diet (415 ml of 13,000 ppm gallic acid equivalent (GAE) for a final concentration of 5.4 g GAE per kg diet or 2.7 g per 2000 kcal (daily human equivalent)) (Table 1). The composition of PFJ was as described previously [99], with major phenolic components being three caffeoylshikimic acid isomers and *p*-hydroxybenzoic acid. Body weight was assessed throughout the feeding period, as were food (in g/d and kJ/d) and fluid intakes. After 4 weeks, random and fasting blood glucose levels were assessed, along with terminal organ weights, plasma lipids and insulin. All experiments and procedures were approved by the Brandeis University Institutional Animal Care and Use Committee.

**Table 1:** Composition of high-carbohydrate diet

Component	Amount (g/kg)
% En	
Carbohydrate	70
Fat	10
Protein	20
En (kJ/g)	16.7
Ingredients (g/kg)	
Casein	100
Lactalbumin	100
Dextrose	350
Corn starch	288 (+60 with gel)^a^
Fat	44
Butter (g of fat)	8
Tallow	15
Soybean oil	23
Mineral mix^b^	44
Vitamin mix^c^	11
Choline chloride	3
Cholesterol	0.6

^a^60 g corn starch was added to 800 ml water to form a gel or added to 375 ml water + 415 ml PFJ (13,000 ppm GAE for a final concentration of 5.4 g GAE per kg diet or 2.7 g per 2000 kcal)

^b^Ausman-Hayes salt mix. Mineral mix contained the following (g/kg mix): magnesium oxide, 320; calcium carbonate, 290.5; potassium phosphate dibasic, 312.2; calcium phosphate dibasic, 72.6; magnesium sulphate, 98.7; sodium chloride, 162.4; ferric citrate, 26.6; potassium iodide, 0.77; manganese sulphate, 3.66; zinc chloride, 0.24; cupric sulphate, 0.29; chromium acetate, 0.044; sodium selenite, 0.004

^c^Hayes-Cathcart vitamin mix. Vitamin mix contained the following (g/kg mix): d-α-tocopheryl acetate (500 IU/g), 15; inositol, 5; niacin, 3; calcium pantothenate, 1.6; retinyl palmitate (500,000 IU/g), 1.5; cholecalciferol (400,000 IU/g), 0.1; menadione, 0.2; biotin, 0.02; folic acid, 0.2; riboflavin, 0.7; thiamin, 0.6; pyridoxine HCl, 0.7; cyanocobalamin, 0.001; dextrin, 972

### Food efficiency

Food efficiency was calculated by dividing body weight gain (in g/d) by caloric intake (in kJ/d) and multiplying the result by 1000. Results represent the body weight gained (g) per 1000 kJ consumed.

### Random and fasting blood glucose

Blood glucose was measured in O_2_/CO_2_-anaesthetised NRs from a drop of tail blood, obtained by lancet puncture of the lateral tail vein using an Elite XL glucometer (Bayer Co., Elhart, IN). Random blood glucose (RBG) was assessed in non-fasted NRs between 9 and 10 am on non-feeding days (semi-purified diets were replenished three times per week). Fasting blood glucose (FBG) was measured at about 9 to 10 am after 16 h of overnight food deprivation.

### Terminal organ weights

Organs (livers, kidneys, caecum and adipose) were weighed after excision, and their weights (in g) were divided by the terminal body weight (in g) to obtain a percentage. The livers were snap-frozen in liquid nitrogen and stored at −80 °C until the total RNA extraction process for gene expression analysis. The relative carcass weight (as percentage body mass) was determined by weighing lean body mass (after exsanguination and excision of all organs) and dividing it by the terminal body weight (in g). Carcass weight was included as an indicator of muscle growth. Body length (nose to base of tail, in cm) was also included as a parameter of growth.

### Plasma biochemical measurements

Plasma triacylglycerol (TG) and total cholesterol (TC) were determined spectrophotometrically using Infinity^TM^ kits (Thermo Fisher Scientific Inc., Middletown, VA, TG ref # TR22421, TC ref # TR13421). Plasma insulin was determined with an ELISA kit for rat/mouse insulin (Linco Research, EMD Millipore, Billerica, MA, ref # EZRMI-13K), according to the manufacturer’s protocol.

### Statistical analyses

Statistical analyses on physiological and biochemical parameters were performed using the Super ANOVA statistical software (Abacus Concepts, Inc., Berkeley, CA). Two-tailed unpaired Student’s *t* test was performed, and differences with *p* values of less than 0.05 were considered statistically significant.

### Microarray gene expression analysis

Total RNA isolation from frozen NR livers was conducted using the RNeasy Plus Mini Kit (Qiagen, Inc., Valencia, CA) and QIAshredder homogenisers (Qiagen, Inc., Valencia, CA), preceded by grinding in liquid nitrogen using mortars and pestles. The total RNA samples obtained were subjected to NanoDrop 1000A Spectrophotometer (Thermo Fisher Scientific, Waltham, MA) measurement for yield and purity assessment. Integrity of the total RNA samples was then assessed using the Agilent 2100 Bioanalyzer (Agilent Technologies, Santa Clara, CA) and Agilent RNA 6000 Nano Chip Assay Kit (Agilent Technologies, Santa Clara, CA).

Amplification of total RNA samples which were of high yield, purity and integrity was performed using the Illumina TotalPrep RNA Amplification Kit (Ambion, Inc., Austin, TX). The complementary ribonucleic acid (cRNA) produced was then hybridised to the Illumina MouseRef-8 Version 2 Expression BeadChip (Illumina, Inc., San Diego, CA), using the Direct Hybridization Kit (Illumina, Inc., San Diego, CA). As each MouseRef-8 BeadChip enables the interrogation of eight samples in parallel, a total of eight cRNA samples were used in the microarray experiment, by selecting four total RNA samples with the highest RNA integrity numbers and 28S/18S ribosomal RNA (rRNA) ratios within each condition. Microarray hybridisation, washing and scanning were conducted according to the manufacturer’s instructions. The raw gene expression data obtained are available at Gene Expression Omnibus [33] (accession number: GSE64901).

Quality control of the hybridisation, microarray raw data extraction and initial analysis were performed using the Illumina BeadStudio software (Illumina, San Diego, CA). Outlier samples were also removed via hierarchical clustering analysis provided by the Illumina GenomeStudio software, via different distance metrics including correlation, absolute correlation, Manhattan and Euclidean distance metrics. Gene expression values were normalised using the rank invariant method, and genes which had a detection level of more than 0.99 in either the control or treatment samples were considered significantly detected.

To filter the data for genes which changed significantly in terms of statistics, the Illumina Custom error model was used and genes were considered significantly changed at a differential score of more than 13, which was equivalent to a *p* value <0.05. Two-way (gene and sample) hierarchical clustering of the significant genes was then performed using the TIGR MeV software to ensure that the replicates of each condition were clustered to each other. The Euclidean distance metric and average linkage method were used to carry out the hierarchical clustering analysis. The genes and their corresponding data were then exported into the Microsoft Excel software for further analysis. To calculate fold changes, an arbitrary value of 10 was given to expression values which were less than 10. Fold changes were then calculated by dividing the mean values of signal Y (treatment) with those of signal X (control) if the genes were up-regulated and vice versa if the genes were down-regulated.

Changes in biological pathways and gene ontologies (biological processes) were then assessed via functional enrichment analysis, using the GO-Elite software. The GO-Elite software ranks WikiPathways [58, 92] and gene ontologies based on the hypergeometric distribution. WikiPathways and gene ontologies which had permuted *p* values of less than 0.05, numbers of genes changed of more than or equal to 2 and *Z* scores of more than 2 were considered significantly changed. In this study, up-regulated and down-regulated genes were analysed separately in the functional enrichment analysis but were viewed together in each WikiPathway, using the PathVisio software [122]. For each of these WikiPathways, boxes coloured yellow indicate genes which were up-regulated while those coloured blue indicate genes which were down-regulated. The fold changes of the genes were indicated next to their boxes.

Changes in regulatory networks were also analysed through the use of the Ingenuity Pathways Analysis software (Ingenuity® Systems, Redwood City, CA). A network is a graphical representation of the molecular relationships between genes or gene products. Genes or gene products were represented as nodes, and the biological relationship between two nodes was represented as an edge (line). The intensity of the node colour indicates the degree of up-regulation (red) or down-regulation (green). Nodes were displayed using various shapes that represented the functional class of the gene product. Edges were displayed with various labels that described the nature of the relationship between the nodes.

### Real-time qRT-PCR validation

Two-step real-time quantitative reverse transcription-polymerase chain reaction (qRT-PCR) was conducted using TaqMan Gene Expression Assays (Applied Biosystems, Foster City, CA) to validate the microarray data obtained. This was performed on six differentially expressed target genes of interest (Table 2), which were selected based on the microarray data analysis performed. The same aliquots of total RNA samples used in the microarray experiments were utilised for this analysis. Primer and probe sets for the selected genes were obtained from the ABI Inventoried Assays-On-Demand (Applied Biosystems, Foster City, CA).

**Table 2:** Genes selected for the real-time qRT-PCR validation experiment

Symbol	Definition	Accession	Assay ID
Target genes			
*Apoc1*	*Apolipoprotein C-I*	NM_007469	Mm00431816_m1
*Apoc3*	*Apolipoprotein C-III*	NM_023114	Mm00445670_m1
*Map3k11*	*Mitogen-activated protein kinase kinase kinase 11*	NM_022012	Mm00491529_m1
*Map3k2*	*Mitogen-activated protein kinase kinase kinase 2*	NM_011946	Mm00442451_m1
*Pik3r3*	*Phosphatidylinositol 3-kinase, regulatory subunit, polypeptide 3 (p55)*	NM_181585	Mm00725026_m1
*Stxbp2*	*Syntaxin binding protein 2*	NM_011503	Mm00441589_m1
Reference genes			
*Cct6a*	*Chaperonin containing Tcp1, subunit 6a (zeta)*	NM_009838	Mm00486818_m1
*Hpd*	*4-hydroxyphenylpyruvic acid dioxygenase*	NM_008277	Mm00801734_m1
*Nipbl*	*Nipped-B homologue (Drosophila)*	NM_027707	Mm01297452_m1
*Trim39*	*Tripartite motif-containing 39*	NM_178281	Mm01273530_m1

The six target genes were selected based on their functional significance, their statistical significance, their presence as single splice transcripts in microarrays and their availability as Taqman assays designed across splice junctions. From the microarray data obtained, four candidate reference genes were also chosen to be tested for expression stability across the groups, with the three most stable ones being finally selected for relative quantification of the target genes

Briefly, reverse transcription to generate first-strand complementary deoxyribonucleic acid (cDNA) from total RNA was conducted using the High-Capacity cDNA Reverse Transcription Kit (Applied Biosystems, Foster City, CA). Real-time PCR was then performed on the first-strand cDNA generated using a 25 μL reaction volume in an Applied Biosystems 7000 Real-Time PCR System (Applied Biosystems, Foster City, CA) using the following conditions: 50 °C, 2 min, 1 cycle; 95 °C, 10 min, 1 cycle; 95 °C, 15 s and 60 °C, 1 min, 40 cycles. For gene expression measurements, reactions for each biological replicate and non-template control (NTC) were performed in duplicates. For amplification efficiency determination, reactions were performed in triplicates.

Quality control of the replicates used, real-time qRT-PCR data extraction and initial analysis were conducted using the 7000 Sequence Detection System software (Applied Biosystems, Foster City, CA). A manual threshold of 0.6000 and an auto baseline were applied in order to obtain the threshold cycle (Ct) for each measurement taken. The threshold was chosen as it intersected the exponential phase of the amplification plots [19]. The criteria for quality control of the data obtained include ∆Ct less than 0.5 between technical replicates and ∆Ct more than 5.0 between samples and NTCs [86].

Relative quantification of the target genes of interest was performed using the qBase 1.3.5 software (Center for Medical Genetics, Ghent University Hospital, Ghent, Belgium) [48], which takes into account the calculations of amplification efficiencies and multiple housekeeping genes. Expression levels of target genes were normalised to the geometric mean of the three most stable reference genes, selected out of the four tested (Table 2). Stability of these reference genes was assessed using the geNorm 3.5 software (Center for Medical Genetics, Ghent University Hospital, Ghent, Belgium) [123].

## Results

### Physiological and biochemical parameters

NRs fed the PFJ-supplemented diet consumed about 15 % fewer calories (*p* < 0.05) than control rats and were associated with significantly lower body weights (*p* < 0.05) (Table 3). Fluid intake did not significantly differ between the two groups. NRs in the PFJ group had less adipose tissue (*p* < 0.05) and a tendency for greater carcass weight (an indicator of lean body mass) and food efficiency. Their caeca were heavier too (*p* < 0.05) compared to the control group. NRs in the PFJ group had significantly lower levels of RBG (*p* < 0.05) and plasma TG (*p* < 0.05) compared to the control group, whereas no significant differences in overnight FBG were observed. Although TC in the PFJ group was slightly greater than that in the control group, it was not significant (*p* > 0.05). Insulin levels also did not differ between the two groups. Liver and kidney weights as percentages of body weights were similar between groups.

**Table 3:** Diabetes assessment parameters of 3-week-old male NRs fed either a high-carbohydrate diet only or a high-carbohydrate diet supplemented with PFJ for 4 weeks

Group	Control		PFJ	
	(*n = 8*)		(*n = 8*)	
	Mean	SD	Mean	SD
Body weight (g)				
Initial (3 weeks old)	37	7	35	8
After 4 weeks	77^a^	8	70^a^	10
Food intake				
g/d	8^a^	1	7^a^	1
kJ/d	134^a^	25	117^a^	13
kcal/d	32^a^	6	28^a^	3
Food efficiency (g body weight gained/1000 kJ)	10.7	1.3	11.1	0.9
Fluid intake (ml/d)	18	7	21	7
Random blood glucose (RBG) (mg/dl)				
After four weeks	241^a^	133	128^a^	121
Fasting blood glucose (FBG) (mg/dl)				
After four weeks	77	38	70	22
Terminal organ weight (% body weight)				
Liver	3.6	0.6	3.6	0.5
Kidneys	0.8	0.2	0.9	0.2
Caecum	1.4^a^	0.4	1.9^a^	0.6
Adipose				
Epididymal	2.9^a^	0.5	2.4^a^	0.8
Perirenal	1.4^a^	0.4	1.1^a^	0.4
Brown fat	1.7^a^	0.2	1.5^a^	0.3
Total fat	6.0^a^	0.8	5.1^a^	1.1
Carcass	73	2	75	5
Body length (cm)	12.9^a^	0.4	12.4^a^	0.7
Plasma lipids (mmol/l)				
Total cholesterol (TC)	3.9	1.3	4.7	2.8
Triacylglycerol (TG)	2.8^a^	1.3	1.9^a^	0.5
Insulin (pmol/l)	0.6	0.3	0.6	0.4

Values sharing a common superscript are significantly different from each other (*p* < 0.05) by two-tailed unpaired Student’s *t* test

### Microarray gene expression

Analysis of microarray gene expression of the NR livers revealed that 71 genes were up-regulated, while 108 genes were down-regulated in the PFJ group compared to the control group (Table 4). A few apolipoprotein genes, including *Apoa1*, *Apoa2*, *Apoc1* and *Apoc3*, were up-regulated in the PFJ group. Several cytochrome P450 genes involved in phase I detoxification, such as *Cyp1a2*, *Cyp2c67*, *Cyp2e1* and *Cyp4f14*, were also up-regulated. Three phase II detoxification genes, i.e. *Ugt2b36*, *Cat* and *Gsto2*, were up-regulated as well. On the other hand, genes down-regulated in the PFJ group include those involved in the insulin-signalling pathway, such as phosphatidylinositol kinases, *Pik3r3* and *Pi4ka*, as well as mitogen-activated protein triple kinases, *Map3k2* and *Map3k11*. Two genes related to fibrosis induction, *Pcolce* and *Plod2*, were also down-regulated in the PFJ group.

**Table 4:** List of genes significantly regulated by PFJ

Symbol	Definition	Differential score	Fold change
Up-regulated genes			
*Sds*	Serine dehydratase	51.92	4.95
*Plekhb1*	Pleckstrin homology domain containing, family B (evectins) member 1	45.01	3.66
*Npc1l1*	Niemann-Pick C1-like 1	30.36	7.41
*EG240549*	Predicted gene, EG240549	25.98	3.13
*F7*	Coagulation factor VII	23.44	3.08
*Ecm1*	Extracellular matrix protein 1	23.18	2.32
*Enpp2*	Ectonucleotide pyrophosphatase/phosphodiesterase 2	21.76	2.50
*Ugt2b36*	UDP glucuronosyltransferase 2 family, polypeptide B36	21.09	2.98
*Hdac3*	Histone deacetylase 3	20.37	2.93
*Cspg5*	Chondroitin sulphate proteoglycan 5	20.27	2.05
*Cyp2c67*	Cytochrome P450, family 2, subfamily c, polypeptide 67	20.06	14.08
*Specc1l*	SPECC1-like	20.05	1.83
*Cps1*	Carbamoyl-phosphate synthetase 1, nuclear gene encoding mitochondrial protein XM_993466	19.42	2.78
*Hbb-b1*	Haemoglobin, beta adult major chain	19.19	2.14
*Tnrc6a*	Trinucleotide repeat containing 6a	19.03	1.58
*Rps7*	Ribosomal protein S7	18.21	1.76
*Apoc1*	Apolipoprotein C-I	17.47	13.49
*Cyp2e1*	Cytochrome P450, family 2, subfamily e, polypeptide 1	16.93	2.33
*Ifrd1*	Interferon-related developmental regulator 1	16.84	1.94
*Mup2*	Major urinary protein 2, transcript variant 1	16.51	197.62
*Rpn2*	Ribophorin II	16.41	2.07
*Asl*	Argininosuccinate lyase	16.33	1.85
*Ptprt*	Protein tyrosine phosphatase, receptor type, T	16.06	2.78
*Bcdo2*	Beta-carotene 9', 10'-dioxygenase 2	16.00	2.43
*Zfhx2*	Zinc finger homeobox 2	15.95	1.77
*Mthfd1*	Methylenetetrahydrofolate dehydrogenase (NADP+ dependent), methenyltetrahydrofolate cyclohydrolase, formyltetrahydrofolate synthase	15.95	1.54
*Rnf215*	Ring finger protein 215	15.91	1.63
*Gne*	Glucosamine	15.82	2.54
*Cyp4f14*	Cytochrome P450, family 4, subfamily f, polypeptide 14	15.60	2.55
*Zxda*	Zinc finger, X-linked, duplicated A	15.35	1.51
*Nat1*	*N*-acetyltransferase 1 (arylamine *N*-acetyltransferase)	15.26	2.05
*Cat*	Catalase	15.23	2.84
*Tyms-ps*	Thymidylate synthase, pseudogene	15.16	1.69
*F5*	Coagulation factor V	15.12	2.38
*Fbxo7*	F-box only protein 7	14.96	1.71
*Apoa2*	Apolipoprotein A-II	14.91	2.67
*Hagh*	Hydroxyacyl glutathione hydrolase	14.87	1.64
*Alas1*	Aminolevulinic acid synthase 1	14.80	10.33
*Inmt*	Indolethylamine *N*-methyltransferase	14.79	2.62
*620807.00*	Predicted gene, 620807	14.78	106.29
*Hsd17b10*	Hydroxysteroid (17-beta) dehydrogenase 10, nuclear gene encoding mitochondrial protein	14.75	2.30
*Nr1i3*	Nuclear receptor subfamily 1, group I, member 3	14.63	2.02
*Nit2*	Nitrilase family, member 2	14.58	1.99
*Tbc1d15*	TBC1 domain family, member 15	14.57	1.71
*Apoc3*	Apolipoprotein C-III	14.56	2.77
*ORF61*	Open reading frame 61	14.51	1.54
*Ephx1*	Epoxide hydrolase 1, microsomal	14.36	2.98
*Serpina1d*	Serine (or cysteine) peptidase inhibitor, clade A, member 1d	14.26	13.47
*Stab1*	Stabilin 1	14.19	2.00
*Ifitm2*	Interferon induced transmembrane protein 2	14.05	1.55
*Hmgcs2*	3-hydroxy-3-methylglutaryl-Coenzyme A synthase 2, nuclear gene encoding mitochondrial protein	14.03	5.41
*Serpina1b*	Serine (or cysteine) preptidase inhibitor, clade A, member 1b	14.03	13.48
*Tmem132e*	Transmembrane protein 132E	13.98	1.99
*Syvn1*	Synovial apoptosis inhibitor 1, synoviolin	13.97	1.78
*Cyp1a2*	Cytochrome P450, family 1, subfamily a, polypeptide 2	13.92	2.38
*Reln*	Reelin	13.90	2.44
*Fzd7*	Frizzled homologue 7 (*Drosophila*)	13.87	1.96
*F13b*	Coagulation factor XIII, beta subunit	13.83	2.30
*Rpl36al*	Ribosomal protein l36a-like	13.73	1.79
*Klkb1*	Kallikrein B, plasma 1	13.72	2.29
*Sdf2*	Stromal cell derived factor 2	13.53	1.44
*3110049J23Rik*	RIKEN cDNA 3110049 J23 gene	13.44	1.70
*2810004N20Rik*	RIKEN cDNA 2810004 N20 gene	13.40	1.74
*Rxrg*	Retinoid X receptor gamma	13.36	2.29
*Ces3*	Carboxylesterase 3	13.20	4.77
*Sec16b*	SEC16 homologue B (*Saccharomyces cerevisiae*)	13.20	2.31
*Gsto2*	Glutathione S-transferase omega 2	13.17	2.33
*5830404H04Rik*	RIKEN cDNA 5830404H04 gene	13.14	1.90
*Creld1*	Cysteine-rich with EGF-like domains 1	13.13	1.46
*Mat1a*	Methionine adenosyltransferase I, alpha	13.04	14.39
*Apoa1*	Apolipoprotein A-I	13.02	25.82
Down-regulated genes			
*St3gal6*	ST3 beta-galactoside alpha-2,3-sialyltransferase 6	−13.09	−21.14
*Btbd3*	BTB (POZ) domain containing 3	−13.12	−2.51
*Wbp2*	WW domain binding protein 2	−13.12	−1.47
*LOC100045542*	Predicted: similar to FERMRhoGEF (Arhgef) and pleckstrin domain protein 1	−13.14	−3.04
*Shmt2*	Serine hydroxymethyltransferase 2 (mitochondrial), nuclear gene encoding mitochondrial protein	−13.19	−1.69
*Clptm1l*	CLPTM1-like	−13.24	−1.48
*Cox10*	COX10 homologue, cytochrome c oxidase assembly protein, heme A: farnesyltransferase (yeast), nuclear gene encoding mitochondrial protein	−13.26	−1.54
*Gpr107*	G protein-coupled receptor 107	−13.27	−1.82
*Dnajc10*	Dnaj (Hsp40) homologue, subfamily C, member 10	−13.29	−1.55
*Plod2*	Procollagen-lysine, 2-oxoglutarate 5-dioxygenase 2	−13.29	−2.74
*Magee1*	Melanoma antigen, family E, 1	−13.31	−4.06
*Ppp1r16a*	Protein phosphatase 1, regulatory (inhibitor) subunit 16A	−13.35	−2.19
*Prkcbp1*	Protein kinase C binding protein 1	−13.36	−2.04
*Map3k11*	Mitogen-activated protein kinase kinase kinase 11	−13.37	−1.48
*Marcks*	Myristoylated alanine rich protein kinase C substrate	−13.38	−1.47
*Tex9*	Testis expressed gene 9	−13.39	−2.46
*Cog1*	Component of oligomeric golgi complex 1	−13.40	−1.51
*Slc39a13*	Solute carrier family 39 (metal ion transporter), member 13	−13.40	−2.66
*Fam110b*	Family with sequence similarity 110, member B	−13.43	−3.23
*Cox6b1*	Cytochrome c oxidase, subunit VIb polypeptide 1	−13.44	−1.45
*Stxbp2*	Syntaxin binding protein 2	−13.45	−1.68
*Ino80b*	INO80 complex subunit B	−13.45	−2.52
*Nap1l4*	Nucleosome assembly protein 1-like 4	−13.45	−1.55
*Flii*	Flightless I homologue (*Drosophila*)	−13.47	−1.63
*Ahdc1*	AT hook, DNA binding motif, containing 1	−13.55	−1.63
*Nol5a*	Nucleolar protein 5A	−13.69	−1.52
*2400001E08Rik*	RIKEN cDNA 2400001E08 gene	−13.75	−1.77
*Prmt5*	Protein arginine N-methyltransferase 5	−13.77	−1.79
*Tinagl*	Tubulointerstitial nephritis antigen-like	−13.78	−3.14
*Parl*	Presenilin associated, rhomboid-like	−13.84	−1.51
*Zmat5*	Zinc finger, matrin type 5	−13.85	−1.86
*Calm3*	Calmodulin 3	−13.86	−2.15
*Ak3l1*	Adenylate kinase 3-like 1, nuclear gene encoding mitochondrial protein	−13.86	−1.49
*2700087H15Rik*	RIKEN cDNA 2700087H15 gene	−13.93	−1.54
*Grit*	RHOGTPase-activating protein	−13.95	−2.23
*X99384*	cDNA sequence X99384	−13.96	−1.77
*Ddx27*	DEAD (Asp-Glu-Ala-Asp) box polypeptide 27	−13.97	−2.09
*Zfp313*	Zinc finger protein 313	−13.98	−1.53
*D15Wsu169e*	DNA segment, Chr 15, Wayne State University 169, expressed	−14.02	−4.17
*Zer1*	Zer-1 homologue (*Caenorhabditis elegans*)	−14.03	−2.01
*Snapc2*	Small nuclear RNA activating complex, polypeptide 2	−14.05	−2.04
*Dock1*	Dedicator of cytokinesis 1	−14.19	−1.91
*Pak4*	P21 (CDKN1A)-activated kinase 4	−14.21	−1.51
*Arl2*	ADP-ribosylation factor-like 2	−14.22	−6.24
*Pcolce*	Procollagen C-endopeptidase enhancer protein	−14.24	−2.15
*1110018G07Rik*	RIKEN cDNA 1110018G07 gene	−14.28	−1.61
*2610528J11Rik*	RIKEN cDNA 2610528J11 gene	−14.29	−2.35
*Akp2*	Alkaline phosphatase 2, liver	−14.31	−2.72
*Mapre1*	Microtubule-associated protein, RP/EB family, member 1	−14.35	−1.56
*Tmem138*	Transmembrane protein 138	−14.36	−2.51
*Pacs2*	Phosphofurin acidic cluster sorting protein 2	−14.41	−1.70
*LOC100047173*	PREDICTED: similar to synaptotagmin-like 1	−14.41	−3.34
*Ano10*	Anoctamin 10	−14.47	−5.94
*Vasn*	Vasorin	−14.48	−1.65
*Cml4*	Camello-like 4	−14.50	−3.02
*Clcn3*	Chloride channel 3, transcript variant a	−14.50	−1.73
*Pik3r3*	Phosphatidylinositol 3-kinase, regulatory subunit, polypeptide 3 (p55)	−14.54	−4.60
*Timp1*	TIMP metallopeptidase inhibitor 1	−14.61	−1.60
*Fbxl15*	F-box and leucine-rich repeat protein 15	−14.65	−1.59
*Npc2*	Niemann-Pick disease, type C2	−14.68	−1.60
*Mrps33*	Mitochondrial ribosomal protein S33, nuclear gene encoding mitochondrial protein, transcript variant 2	−14.73	−1.65
*Pgam5*	Phosphoglycerate mutase family member 5	−14.73	−1.84
*2310005N01Rik*	RIKEN cDNA 2310005N01 gene	−14.79	−2.67
*Ctdspl*	CTD (carboxy-terminal domain, RNA polymerase II, polypeptide A) small phosphatase-like	−14.83	−2.49
*LOC100046039*	PREDICTED: similar to histone deacetylase HD1	−14.85	−2.29
*Gnptab*	*N*-acetylglucosamine-1-phosphate transferase, alpha and beta subunits	−14.93	−1.90
*Tbc1d14*	TBC1 domain family, member 14	−15.03	−2.88
*Cyr61*	Cysteine-rich protein 61	−15.07	−4.37
*Gdpd1*	Glycerophosphodiester phosphodiesterase domain containing 1	−15.11	−1.58
*2310022B05Rik*	RIKEN cDNA 2310022B05 gene	−15.16	−1.53
*Asna1*	Arsa arsenite transporter, ATP-binding, homologue 1 (bacterial)	−15.16	−1.66
*Tcf4*	Transcription factor 4, transcript variant 1	−15.17	−2.10
*Vps26b*	Vacuolar protein sorting 26 homologue B (yeast)	−15.43	−1.57
*Nf2*	Neurofibromatosis 2	−15.54	−2.64
*LOC192758*	Similar to hypothetical protein MGC39650	−15.63	−3.10
*Drg2*	Developmentally regulated GTP binding protein 2	−15.66	−1.74
*Iqgap1*	IQ motif containing GTPase activating protein 1	−15.89	−1.73
*Nrp1*	Neuropilin 1	−16.05	−2.33
*Tbc1d13*	TBC1 domain family, member 13	−16.13	−3.24
*2310003P10Rik*	RIKEN cDNA 2310003P10 gene	−16.15	−3.82
*Trim28*	Tripartite motif protein 28	−16.18	−1.79
*Tlr2*	Toll-like receptor 2	−16.41	−2.26
*0910001L09Rik*	RIKEN cDNA 0910001L09 gene	−16.42	−2.15
*B930041F14Rik*	RIKEN cDNA B930041F14 gene	−16.74	−2.44
*Nup93*	Nucleoporin 93 kDa	−16.93	−2.22
*Lphn1*	Latrophilin 1	−17.11	−2.08
*Odz4*	Odd Oz/ten-m homologue 4 (*Drosophila*)	−17.13	−4.10
*Gnai2*	Guanine nucleotide binding protein, alpha inhibiting 2	−17.14	−2.08
*Cyp4f13*	Cytochrome P450, family 4, subfamily f, polypeptide 13	−17.16	−4.76
*Aacs*	Acetoacetyl-coa synthetase	−17.26	−1.62
*Smarca4*	SWI/SNF related, matrix associated, actin dependent regulator of chromatin, subfamily a, member 4	−17.42	−1.89
*Gatad2b*	GATA zinc finger domain containing 2B	−17.46	−2.31
*Actr1b*	ARP1 actin-related protein 1 homologue B, centractin beta (yeast)	−17.87	−1.74
*Neo1*	Neogenin 1	−17.93	−2.00
*Meis2*	Meis homeobox 2, transcript variant 2	−18.31	−1.91
*Serpinh1*	Serine (or cysteine) peptidase inhibitor, clade H, member 1	−18.44	−9.72
*Cc2d2a*	Coiled-coil and C2 domain containing 2A	−18.44	−2.28
*Vdac1*	Voltage-dependent anion channel 1	−18.88	−1.65
*Picalm*	Phosphatidylinositol binding clathrin assembly protein	−19.13	−1.73
*Ankrd24*	Ankyrin repeat domain 24	−19.20	−6.41
*Pi4ka*	Phosphatidylinositol 4-kinase, catalytic, alpha polypeptide	−19.52	−2.19
*Map3k2*	Mitogen-activated protein kinase kinase kinase 2	−19.53	−3.51
*1700029G01Rik*	RIKEN cDNA 1700029G01 gene	−19.78	−2.21
*Atn1*	Atrophin 1	−21.42	−1.86
*Itprip*	Inositol 1,4,5-triphosphate receptor interacting protein	−22.26	−6.38
*Gadd45g*	Growth arrest and DNA-damage-inducible 45 gamma	−23.65	−2.44
*Ly6e*	Lymphocyte antigen 6 complex, locus E	−23.91	−2.53
*Ctcfl*	CCCTC-binding factor (zinc finger protein)-like	−27.64	−2.21

**Fig. 1:**
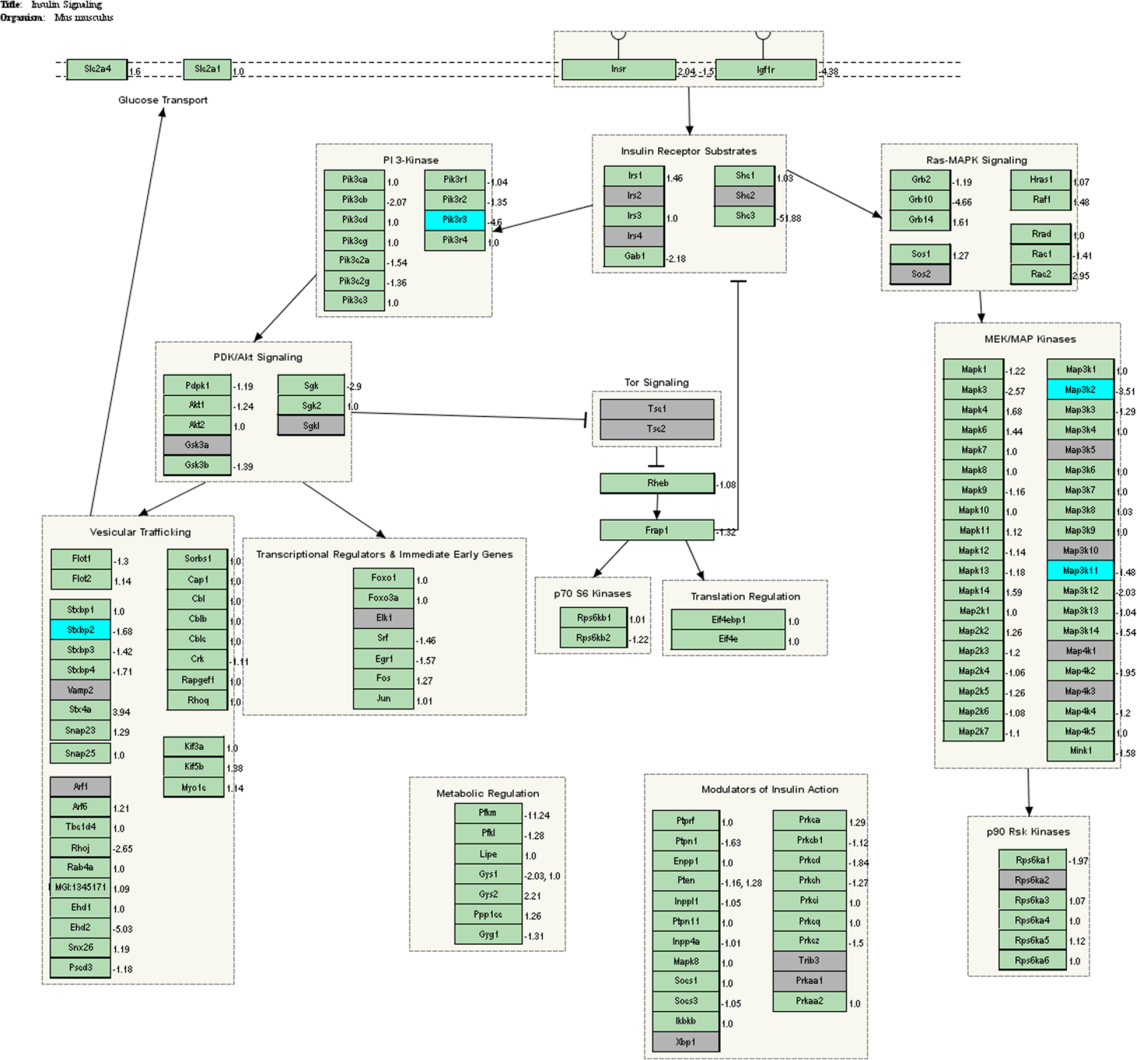
Insulin-signalling pathway related genes down-regulated by PFJ in the liver of NRs

Functional enrichment analysis showed that various biological pathways (Table 5) and gene ontologies (biological processes) (Table 6) were differentially regulated in NRs given PFJ compared to controls. Among WikiPathways up-regulated by PFJ were those of tryptophan metabolism, methylation, fatty acid omega oxidation, nuclear receptors in lipid metabolism and toxicity, complement and coagulation cascades, urea cycle and metabolism of amino groups, retinol metabolism, metapathway biotransformation, one-carbon metabolism and nuclear receptors, as well as cytochrome P450s. Down-regulated WikiPathways include regulation of actin cytoskeleton, insulin signalling and TNF-alpha NF-κβ signalling. In relation to T2DM, a significant observation was that the insulin-signalling pathway was down-regulated in the PFJ group (Fig. 1).

**Table 5:** List of WikiPathways significantly regulated by PFJ

WikiPathway name	No. changed	% changed	*Z* score	Permuted *p*
Up-regulated WikiPathways				
Tryptophan metabolism:WP79	6	15.3846	9.4735	0.0000
Aflatoxin B1 metabolism:WP1262	2	40.0000	9.1192	0.0005
Methylation:WP1247	2	28.5714	7.6363	0.0000
Fatty acid omega oxidation:WP33	2	28.5714	7.6363	0.0015
Statin pathway (PharmGKB):WP1	3	16.6667	6.9838	0.0000
Blood clotting cascade:WP460	3	15.7895	6.7763	0.0000
Nuclear receptors in lipid metabolism and toxicity:WP431	3	10.0000	5.2068	0.0005
Complement and coagulation cascades:WP449	4	6.8966	4.7841	0.0000
Urea cycle and metabolism of amino groups:WP426	2	11.1111	4.5187	0.0020
Retinol metabolism:WP1259	3	7.6923	4.4328	0.0005
Metapathway biotransformation:WP1251	5	4.4248	3.9468	0.0000
One-carbon metabolism:WP435	2	8.3333	3.7980	0.0040
Nuclear receptors:WP509	2	5.5556	2.9123	0.0075
Cytochrome P450:WP1274	2	5.2632	2.8039	0.0105
Down-Regulated WikiPathways				
Regulation of actin cytoskeleton:WP523	4	3.0534	3.6287	0.0080
Insulin signalling:WP65	4	2.8169	3.4170	0.0110
Endochondral ossification:WP1270	2	3.3898	2.7401	0.0460
TNF-alpha NF-κβ signalling pathway:WP246	3	2.2222	2.4278	0.0430

**Table 6:** List of gene ontologies (biological processes) significantly regulated by PFJ

GO ID	GO name	No. changed	% changed	*Z* score	Permuted *p*
Up-regulated gene ontologies (biological processes)					
GO:0010903	Negative regulation of very-low-density lipoprotein particle remodelling	3	100.0000	26.2353	0.0000
GO:0060192	Negative regulation of lipase activity	4	40.0000	19.0387	0.0000
GO:0033700	Phospholipid efflux	4	33.3333	17.3429	0.0000
GO:0060416	Response to growth hormone stimulus	4	30.7692	16.6448	0.0000
GO:0032488	Cdc42 protein signal transduction	2	50.0000	15.0814	0.0000
GO:0046461	Neutral lipid catabolic process	3	33.3333	15.0179	0.0000
GO:0042157	Lipoprotein metabolic process	5	20.0000	14.8938	0.0000
GO:0007494	Midgut development	3	30.0000	14.2268	0.0000
GO:0048261	Negative regulation of receptor-mediated endocytosis	2	40.0000	13.4602	0.0000
GO:0010915	Regulation of very-low-density lipoprotein particle clearance	2	40.0000	13.4602	0.0000
GO:0071825	Protein-lipid complex subunit organisation	4	19.0476	12.9842	0.0000
GO:0015918	Sterol transport	5	14.2857	12.4800	0.0000
GO:0050995	Negative regulation of lipid catabolic process	3	23.0769	12.4241	0.0005
GO:0030300	Regulation of intestinal cholesterol absorption	2	33.3333	12.2609	0.0000
GO:0034381	Plasma lipoprotein particle clearance	3	21.4286	11.9549	0.0000
GO:0008203	Cholesterol metabolic process	7	9.5890	11.9276	0.0000
GO:0034367	Macromolecular complex remodelling	3	20.0000	11.5328	0.0000
GO:0010873	Positive regulation of cholesterol esterification	2	28.5714	11.3268	0.0000
GO:0018904	Organic ether metabolic process	7	8.6420	11.2675	0.0000
GO:0071941	Nitrogen cycle metabolic process	2	22.2222	9.9459	0.0000
GO:0051055	Negative regulation of lipid biosynthetic process	3	13.6364	9.4265	0.0000
GO:0071320	Cellular response to cyclic adenosine monophosphate	2	20.0000	9.4149	0.0005
GO:0042632	Cholesterol homeostasis	4	10.0000	9.2153	0.0000
GO:0071396	Cellular response to lipid	3	13.0435	9.2059	0.0005
GO:0071383	Cellular response to steroid hormone stimulus	5	6.4935	8.1091	0.0000
GO:0006720	Isoprenoid metabolic process	4	7.0175	7.5752	0.0000
GO:0050817	Coagulation	4	5.8824	6.8498	0.0000
GO:0001101	Response to acid	4	5.7971	6.7922	0.0005
GO:0010243	Response to organic nitrogen	5	4.7170	6.7315	0.0000
GO:0044272	Sulphur compound biosynthetic process	3	7.3171	6.7133	0.0015
GO:0017144	Drug metabolic process	2	10.5263	6.6960	0.0045
GO:0033762	Response to glucagon stimulus	3	6.9767	6.5356	0.0005
GO:0044106	Cellular amine metabolic process	9	2.7692	6.4744	0.0000
GO:0043436	Oxoacid metabolic process	12	2.1053	6.1883	0.0000
GO:0033574	Response to testosterone stimulus	2	9.0909	6.1811	0.0030
GO:0009636	Response to toxin	4	4.7619	6.0507	0.0010
GO:0031100	Organ regeneration	3	5.8824	5.9287	0.0010
GO:0042743	Hydrogen peroxide metabolic process	2	8.3333	5.8913	0.0045
GO:0031667	Response to nutrient levels	7	2.7237	5.6322	0.0000
GO:0051262	Protein tetramerisation	3	5.3571	5.6146	0.0020
GO:0031330	Negative regulation of cellular catabolic process	2	7.1429	5.4051	0.0060
GO:0030193	Regulation of blood coagulation	2	6.8966	5.2990	0.0060
GO:0010043	Response to zinc ion	2	6.8966	5.2990	0.0050
GO:0031647	Regulation of protein stability	3	4.8387	5.2867	0.0020
GO:0051384	Response to glucocorticoid stimulus	4	3.3898	4.9035	0.0010
GO:0007623	Circadian rhythm	3	4.2254	4.8711	0.0040
GO:0055114	Oxidation-reduction process	11	1.6129	4.7935	0.0000
GO:0071375	Cellular response to peptide hormone stimulus	4	3.1008	4.6273	0.0035
GO:0006725	Cellular aromatic compound metabolic process	4	2.9851	4.5123	0.0015
GO:0033013	Tetrapyrrole metabolic process	2	5.1282	4.4651	0.0070
GO:0051186	Cofactor metabolic process	5	2.4510	4.4123	0.0005
GO:0033555	Multicellular organismal response to stress	2	4.3478	4.0441	0.0110
GO:0044262	Cellular carbohydrate metabolic process	6	1.8127	3.8583	0.0045
GO:0061061	Muscle structure development	2	3.8462	3.7493	0.0190
GO:0042445	Hormone metabolic process	3	2.7027	3.6494	0.0100
GO:0042493	Response to drug	5	1.8116	3.5138	0.0045
GO:0006730	One-carbon metabolic process	4	1.9802	3.3655	0.0125
GO:0007626	Locomotory behaviour	3	2.3810	3.3384	0.0125
GO:0014070	Response to organic cyclic compound	4	1.9417	3.3146	0.0065
GO:0048513	Organ development	9	1.1704	3.1890	0.0050
GO:0009607	Response to biotic stimulus	6	1.4458	3.1796	0.0090
GO:0009611	Response to wounding	5	1.6026	3.1729	0.0120
GO:0009791	Post-embryonic development	2	2.7778	3.0325	0.0375
GO:0006414	Translational elongation	2	2.6316	2.9216	0.0485
GO:0010466	Negative regulation of peptidase activity	3	1.9868	2.9171	0.0325
GO:0050679	Positive regulation of epithelial cell proliferation	2	2.4096	2.7455	0.0480
GO:0035335	Peptidyl-tyrosine dephosphorylation	2	2.3529	2.6988	0.0380
GO:0034284	Response to monosaccharide stimulus	2	2.3529	2.6988	0.0490
GO:0032989	Cellular component morphogenesis	4	1.4652	2.6156	0.0285
GO:0009967	Positive regulation of signal transduction	5	1.1876	2.3859	0.0305
Down-regulated gene ontologies (biological processes)					
GO:0032006	Regulation of mTORsignalling cascade	2	12.5000	6.1613	0.0040
GO:0031113	Regulation of microtubule polymerisation	2	11.1111	5.7727	0.0070
GO:0001702	Gastrulation with mouth forming second	2	9.0909	5.1559	0.0075
GO:0045216	Cell-cell junction organisation	2	5.2632	3.7231	0.0245
GO:0042632	Cholesterol homeostasis	2	5.0000	3.6045	0.0235
GO:0006793	Phosphorus metabolic process	14	1.4433	3.5098	0.0015
GO:0031214	Biomineral tissue development	2	4.5455	3.3902	0.0280
GO:0002263	Cell activation involved in immune response	2	4.5455	3.3902	0.0320
GO:0042475	Odontogenesis of dentine-containing tooth	2	4.4444	3.3408	0.0315
GO:0032259	Methylation	4	2.5157	3.1405	0.0145
GO:0030155	Regulation of cell adhesion	4	2.4096	3.0318	0.0160
GO:0050790	Regulation of catalytic activity	15	1.2490	3.0295	0.0080
GO:0010243	Response to organic nitrogen	3	2.8302	2.9800	0.0270
GO:0001933	Negative regulation of protein phosphorylation	2	3.6364	2.9179	0.0460
GO:0071841	Cellular component organisation or biogenesis at cellular level	18	1.0508	2.5596	0.0140
GO:0019219	Regulation of nucleobase, nucleoside, nucleotide and nucleic acid metabolic process	21	0.9620	2.3612	0.0210
GO:0008219	Cell death	7	1.3514	2.2489	0.0340

Up-regulated gene ontologies (biological processes) of interest include negative feedback of very-low-density lipoprotein particle remodelling, negative feedback of receptor-mediated endocytosis, negative feedback of very-low-density lipoprotein particle clearance, negative feedback of lipid catabolic process, macromolecular complex remodelling, positive feedback of cholesterol esterification, negative feedback of lipid biosynthetic process, cellular response to lipid, cellular response to steroid hormone stimulus, negative feedback of cellular catabolic process, oxidation-reduction process, cellular response to peptide hormone stimulus and cellular carbohydrate metabolic process, as well as positive feedback of signal transduction. On the other hand, down-regulated gene ontologies (biological processes) of interest include mammalian target of rapamycin (mTOR) signalling cascade, microtubule polymerisation, cell-cell junction organisation, cell activation involved in immune response, methylation, cell adhesion and catalytic activity, as well as negative feedback of protein phosphorylation.

**Fig. 2:**
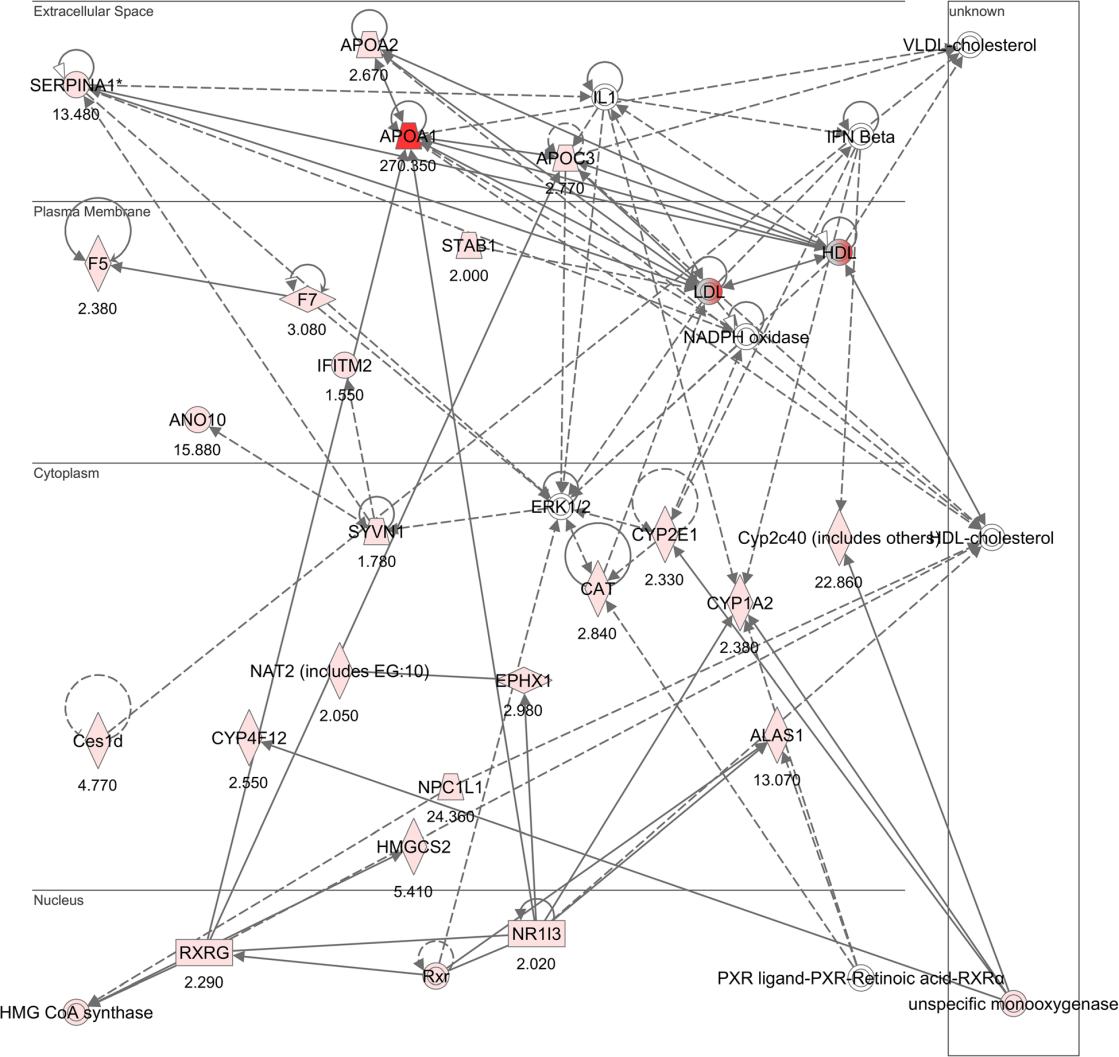
Apolipoprotein genes up-regulated by PFJ in the liver of NRs

Network analysis using the Ingenuity Pathways Analysis software showed that several apolipoproteins including apolipoproteins A1, A2 and C3 were up-regulated by PFJ (Fig. 2). In addition, apolipoprotein C1 was up-regulated as well (Table 4).

### Real-time qRT-PCR validation

**Fig. 3:**
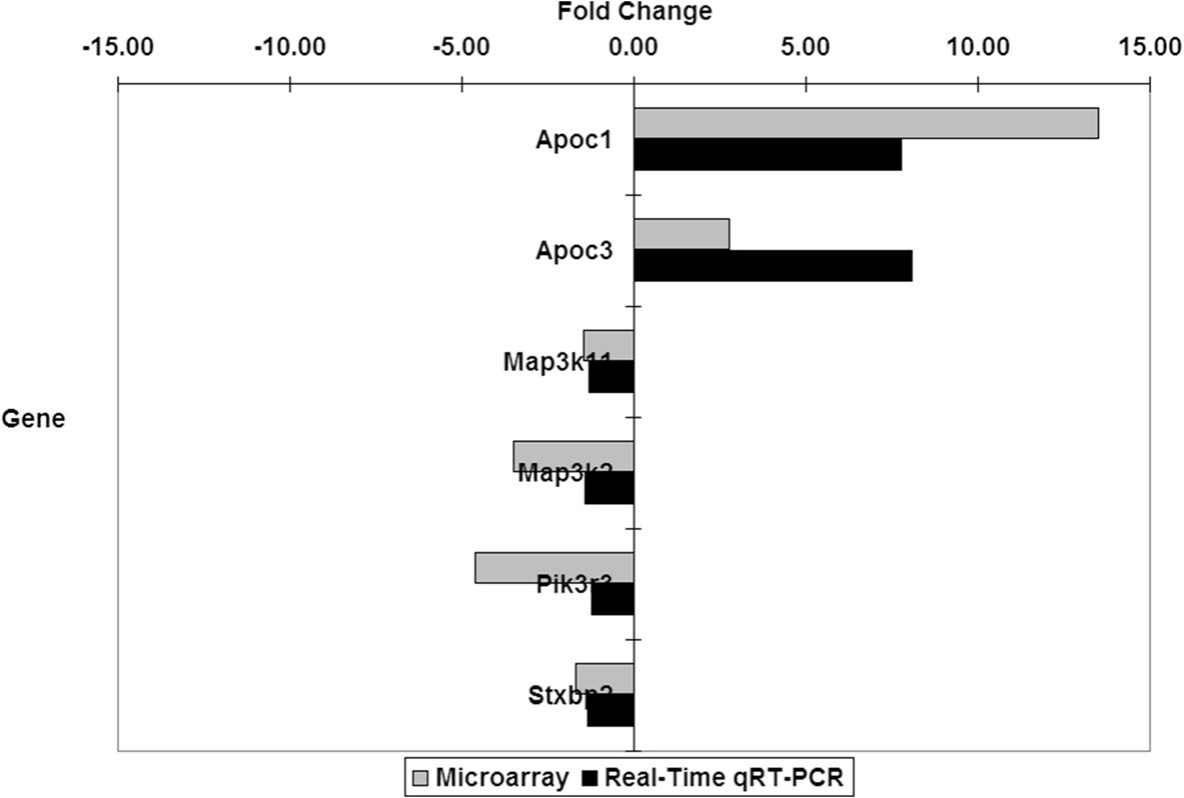
Gene expression fold changes quantified by microarray and real-time qRT-PCR

To confirm the microarray results, the expression levels of six selected target genes were measured using real-time quantitative reverse transcription-polymerase chain reaction (qRT-PCR). From the four selected candidate reference genes tested, analysis using the geNorm 3.5 software [123] showed that *Hpd*, *Nipbl* and *Trim39* were more stable than *Cct6a*. Hence, the former three were selected as the reference genes to normalise the expression values of the target genes. The directions of fold changes of the target genes obtained from the real-time qRT-PCR technique as quantified by the qBase software [48] were comparable to those obtained from the microarray technique (Fig. 3). However, the magnitudes of fold changes obtained using real-time qRT-PCR were consistently lower than those obtained using microarrays.

## Discussion

Rapid economic progress has resulted in lifestyle changes, especially in diet and physical activity. In combination with aging populations, this has resulted in a worldwide epidemic of obesity, T2DM and metabolic syndrome [105]. In the USA, the prevalence of obesity which leads to T2DM and the metabolic syndrome has risen, even as the intake of fat is reduced. This has been referred to as the American Paradox [17], and high-carbohydrate intake has been suggested to be the cause of the problem [9].

Many phenolic-rich extracts have been suggested to be beneficial in preventing or treating T2DM and its related complications. In line with this, we have previously shown that providing PFJ at 1800 mg/L GAE ad libitum as the sole drinking fluid for 17 weeks blocked T2DM and metabolic syndrome progression in 12-week-old male NRs, as evidenced by normalisation of initially elevated blood glucose and plasma lipids [15, 16, 100]. The anti-diabetic effects of PFJ appeared relatively independent of starting age, and no impairment of energy intake or body weight dynamics have been observed in mature NRs, nor were any other toxic effects attributed to it [16]. In addition, PFJ protection against blood glucose elevation has also previously been shown to occur independently of diet (chow or semi-purified, moderate or high carbohydrate), study duration, initial blood glucose or application method [16]. PFJ may thus represent a source for food supplementation or as a nutraceutical having possible anti-diabetic properties.

### PFJ reduced weight gain, adipose tissue, plasma TG and plasma RBG but increased caecum weight

Following the 4-week high-carbohydrate diet challenge in weanling male NRs, the group supplemented with PFJ weighed less and their food intake was significantly lower. However, the carcass (lean mass) and food efficiency tended to be greater for the PFJ group, and they had less adipose tissue. Thus, control rats gained more weight than those in the PFJ group, mostly due to the accumulation of adipose tissue, while PFJ seemed to inhibit appetite and reduce body fat percentage without reducing food efficiency or leading to a decrease in lean body mass. The latter effect is a characteristic of dietary fibres that are fermented by large bowel microbiota [107], and it is noteworthy that the enlarged caeca in rats fed PFJ would be consistent with enhanced fermentation of PFJ components by their large bowel flora. Faster weight gain in male NRs has also been found to enhance T2DM induction in growing rats [15, 21]. As visceral adiposity and hyperlipaemia are two of the risk factors for cardiovascular insults in metabolic syndrome, the reduced body fat percentage and TG levels observed in the PFJ group indicate a beneficial metabolic effect beyond improved blood glucose levels.

The PFJ group also had a significantly lower level of RBG compared to the controls, although no differences were observed in terms of FBG. RBG is an early and more reliable parameter of T2DM than FBG in NRs [14–16, 21, 85]. This is because the correlations between circulating glucose and different markers of T2DM, such as elevations in HbA1c and hypertension, are stronger for RBG than FBG in NRs. In addition, acute cell and organ damage is best reflected by the degree and duration of postprandial hyperglycaemia, thus rendering RBG the best indicator of such damage [15, 16]. The observation that insulin levels were not significantly different between the two groups (*p* > 0.05) indicates that the improved glucose control was due to mechanisms other than increased insulin secretion, such as reduced intestinal glucose absorption or improved insulin sensitivity. As hyperinsulinaemia is one of the first indicators of insulin resistance and a risk factor for the eventual depletion of pancreatic beta cells, this is a crucial observation for the prevention of T2DM, potentially reducing the need for or delaying the onset of insulin therapy or enabling a reduced dose. PFJ thus exerted beneficial metabolic effects, preventing NRs from overconsumption of calories and achieving improved control of plasma glucose and lipids.

As NRs in the present study were fed ad libitum, at least part of the effects ascribed to PFJ could be due to mild caloric restriction caused by reduced food intake. Nevertheless, caloric restriction in the classical sense typically entails a 20–40 % reduction in food consumption relative to normal intake [64], which was not the case here at 15–20 %. Furthermore, we previously found no reduction in food intake or any difference in PFJ protection in older NRs when given artificially sweetened PFJ, suggesting that PFJ protection against diabetes development does not depend on reductions in food consumption [16].

In addition, NRs in the PFJ group had heavier caeca (*p* < 0.05) than the controls. This may be attributed to the presence of fermentable dietary fibres in the PFJ extract that resisted upper gut digestion and reached the caecum (the main site of bacterial fermentation in rodents) where they were fermented by the microbiota. However, the bioactive components in PFJ and/or their derived metabolites may have also played a part in the observed caecum enlargement. In the colon, where microbial glucosidases and glucuronidases are active, phenolic glycosides are intensively metabolised and their metabolites also modify colon parameters, such as short-chain fatty acids, amino acids and vitamins [30]. This is in agreement with the results of others, where increased caecal weight was observed in rats fed diets containing polyphenols [2, 37, 53]. Romo-Vaquero et al. [95] also reported that rosemary extract enriched in the bioactive compound carnosic acid caused caecum enlargement in female Zucker rats. The presence of non-digested materials fermented by large bowel microbiota might have caused the enlarged caeca. The same study also reported that the rosemary extract lowered body weights, serum lipids and insulin levels in the rats and partially attributed this to the inhibition of a pre-duodenal butyrate esterase activity [95]. Thus, the lower adipose tissue content and body weights of the NRs on PFJ may also have been a consequence of the inhibition of specific enzymes in the gut. A pomegranate extract, rich in punicalagin and ellagic acid, also increased caecum size and *Bifidobacterium* in mice [84]. The gut microbiota can modulate host energy metabolism and is thus a significant contributor to the development of obesity and metabolic disorders [130].

### Microarray gene expression analysis revealed down-regulation of the insulin-signalling pathway linked to altered insulin availability

Research on the health effects of plant-based foods will benefit from taking a holistic approach to understand the plethora of effects mediated by a range of bioactive metabolites derived from plant consumption. Thus, the combination of different ‘omics’ profiling techniques in the concept of systems biology, or nutrigenomics as termed in the context of nutrition-related sciences, would be important for this purpose [47]. In the present study, microarrays delineated hepatic gene expression differences between young NRs supplemented with PFJ or not and further confirmed several target genes of interest using real-time qRT-PCR.

In relation to T2DM, the most significant observation from the functional enrichment analysis of the microarray gene expression data was that the insulin-signalling pathway was down-regulated in NRs given PFJ, including genes for mitogen-activated protein triple kinases, *Map3k2* and *Map3k11*, phosphatidylinositol kinases, *Pik3r3* and *Pi4ka*, as well as syntaxin binding protein 2 (*Stxbp2*).

Insulin is essential for appropriate tissue development, growth and maintenance of whole body glucose homeostasis. This hormone is secreted by the β cells of the pancreatic islets of Langerhans in response to increased circulating levels of glucose after a meal. Insulin regulates glucose homeostasis by reducing hepatic glucose output and increasing the rate of glucose uptake primarily into striated muscle and adipose tissues. In these tissues, the clearance of circulating glucose depends on the insulin-stimulated translocation of the facilitative glucose transporter 4 (GLUT4) to the cell surface. Insulin also profoundly affects lipid metabolism by increasing lipid synthesis in liver and adipose tissues, as well as attenuating fatty acid release from TG in fat and muscle cells. Insulin resistance occurs when normal circulating concentrations of the hormone are insufficient to dispose of circulating glucose imposed by glucose-rich diets. In fact, insulin rises dramatically in concert with insulin resistance in the early diabetes of NRs fed high-glycaemic load diets, then falls as diabetes progresses [15].

To assure insulin sensitivity, the circulating hormone must bind to an enzyme that activates its functions, in this case the α-subunit of the insulin receptor embedded in the cell membrane. This binding triggers the tyrosine kinase activity in the β-subunit of the insulin receptor, which further causes phosphorylation of two types of enzymes, mitogen-activated protein kinases (MAPKs) and phosphatidylinositol 3-kinases (PI3Ks), which are responsible for expressing the mitogenic and metabolic actions of insulin, respectively [111]. The activation of MAPKs leads to the completion of mitogenic functions such as cell growth and gene expression, while the activation of PI3Ks leads to important metabolic functions such as synthesis of lipids, proteins and glycogen, as well as cell survival and cell proliferation. Most importantly, the PI3K pathway is responsible for the distribution of glucose for essential cell functions.

#### MAPKs

In our present study, two enzymes involved in the MAPK pathway of insulin signalling, i.e. *Map3k2* and *Map3k11*, were down-regulated in PFJ-supplemented rats. Many studies have causally implicated MAPKs in the development of insulin resistance [96]. Systemic insulin resistance triggers chronic hyperglycaemia, which causes pancreatic β cells to secrete more insulin. In the long term, this adaptation is associated with stress-induced β cell death and leads to insulin deficiency and T2DM. As such, stress mechanisms that trigger insulin resistance are also known to contribute to β cell failure. The majority of studies indicate that prolonged enhanced MAPK signalling is detrimental to insulin sensitivity and β cell function. A growing body of evidence also indicates that MAPKs are involved in physiological metabolic adaptation, the disturbance of which might contribute to metabolic diseases. Thus, although MAPK-dependent signal transduction is required for physiological metabolic adaptation, inappropriate MAPK signalling contributes to the development of T2DM and the metabolic syndrome [41].

#### GLUT4

By definition, insulin resistance is a defect in signal transduction associated with accumulation of diacylglycerol and ceramides [91, 101]. At present, only one class of downstream signalling molecules is confirmed to be essential for insulin-stimulated glucose uptake and GLUT4 translocation, i.e. the class IA PI3Ks [27]. The GLUT4 vesicle, which is responsible for passive diffusion of glucose, binds to PI3Ks after bringing glucose into the cell. PI3Ks isolate the GLUT4 vesicle from the glucose and send the vesicle back to the cell membrane. The glucose that is isolated is then sent to the mitochondria to produce energy as ATP, and excess glucose is stored in the cell as glycogen, which is increased in NRs with T2DM [21]. The binding of insulin to its receptor on the surface of adipose and muscle cells initiates a signalling cascade that alters the trafficking itinerary of GLUT4 thus releasing it from intracellular stores and delivering it to the cell surface [18, 109]. In the absence of insulin, about 95 % of GLUT4 is confined to intracellular compartments. Insulin stimulation results in GLUT4 redistribution from these intracellular stores to the plasma membrane via alterations in membrane trafficking [18, 109]. This insulin-stimulated translocation of GLUT4 from intracellular sites to the plasma membrane is defective in individuals with insulin resistance and T2DM thus providing an impetus to comprehend how this trafficking pathway is controlled [12, 44].

#### PI3Ks

Emerging data indicate that the products of class IA PI3Ks act as both membrane anchors and allosteric regulators, serving to localise and activate downstream enzymes and their protein substrates [106]. Several studies have suggested that the interaction of insulin receptor substrate (IRS) proteins with PI3Ks is necessary for the appropriate activation and/or targeting of the enzyme to a critical intracellular site, including its association with GLUT4 vesicles [91]. Class IA PI3Ks play an essential role in insulin stimulation of glucose transport and metabolism and protein and lipid synthesis, as well as cell growth and differentiation [98].

In terms of molecular structure, class IA PI3Ks are heterodimers consisting of one regulatory and one catalytic subunit, each of which occurs in multiple isoforms [118, 119]. Three mammalian genes, *Pik3r1*, *Pik3r2* and *Pik3r3* encode for the p85α (p85α, p50α and p55α isoforms), p85β and p55γ regulatory subunits, respectively. The family of the catalytic subunits includes p110α, p110β, and p110δ [106]. These are the products of three respective genes, *Pik3ca*, *Pik3cb* and *Pik3cd*. The regulatory subunits of class IA PI3Ks appear to play three important functional roles. They confer stability on the catalytic subunits, induce lipid kinase activity upon insulin stimulation [131] and, in the basal state, inhibit the catalytic activity of the p110 subunits to various degrees [116].

The unique structural domains of the PI3K regulatory subunits and their differential abundances in tissues suggest that they are not entirely redundant and may serve unique purposes. Complete disruption of hepatic *Pik3r1* and *Pik3r2* markedly reduces insulin-stimulated PI3K activity, at least in part by destabilising the catalytic subunits [112]. On the other hand, partial loss of the regulatory subunits of PI3Ks increases insulin sensitivity, and this appears to be related to diminished negative feedback to the IRS proteins [40]. For example, mice with a knockout of the full-length p85α exhibit an up-regulation of the splice variants p50α and p55α in muscle and fat tissues and have increased insulin sensitivity [114]. In addition, p50α/p55α knockout mice exhibit improved insulin sensitivity, lower fat masses and protection against obesity-induced insulin resistance [23]. However, mice with complete deletion of p85α and its short splice variants p50α and p55α die perinatally with liver necrosis and enlarged muscle fibres [38]. Thus, identifying the precise pathways uniquely mediated by these regulatory subunit isoforms remains an important area for further study.

In the present study, the *Pik3r3* gene encoding for the p55γ regulatory subunit of PI3Ks was down-regulated in NRs given PFJ. p55γ is similar in structure to p55α but is expressed at low levels in most tissues [111]. However, the effect of inhibiting or knocking out p55γ, encoded by the *Pik3r3* gene, on insulin sensitivity has not been conclusively determined. Nevertheless, since rats given PFJ had lower levels of RBG (*p* < 0.05) but similar insulin levels compared to NRs in the control group, the down-regulation of the *Pik3r3* gene and the related hepatic insulin-signalling pathway in general suggests that reduced glucose absorption by PFJ lowered the diabetogenic effects of the high-carbohydrate diet and/or enhanced insulin sensitivity, rather than PFJ acting by increasing insulin secretion. This is in accordance with the physiological parameters, as outlined above. The down-regulation of the insulin-signalling pathway could prove beneficial in the long run, as this would protect the pancreas from overproducing insulin and preserve insulin sensitivity in the related target organs, thereby preventing hyperinsulinaemia and hyperglycaemia.

### Down-regulation of hepatic genes involved in fibrotic processes was observed in NRs given PFJ

#### T2DM and hepatic diseases

T2DM and obesity are risk factors for non-alcoholic fatty liver diseases, which include hepatic steatosis (non-alcoholic fatty liver disease or NAFLD), non-alcoholic steatohepatitis (NASH), fibrosis and cirrhosis. Increased insulin resistance and adiposity contribute to the progression from non-alcoholic steatohepatitis to fibrosis through the development of a pro-fibrotic condition in the liver, including increased hepatocellular death, increased generation of reactive oxygen species and an altered cytokine balance [24]. Liver disease is an important cause of death in T2DM, as T2DM is currently the most common cause of liver disease in the USA, including the hepatocellular carcinoma that results from chronic T2DM [115]. The prevalence of T2DM in cirrhosis is 12.3 to 57 % [117].

Incidentally, hepatic steatosis is the most prevalent early lesions in diabetic NRs and is correlated with advancing T2DM, with hepatomegaly and liver discolouration also present macroscopically [70]. A large proportion of male NRs that reach 1 year of age with T2DM also reveal hepatocellular carcinoma in various stages (Kenneth C. Hayes, Brandeis University, MA, personal communication).

#### Collagen accumulation and fibrosis

Organ fibrosis including liver fibrosis is characterised by an excessive accumulation of collagen. Mature collagen cross-links in a variety of connective tissues such as bones, tendons, ligaments and cartilages are formed via the hydroxyallysine route. In contrast, collagen in the skin is mainly cross-linked via the allysine route. In organ fibrosis, an increase in cross-links derived from the hydroxyallysine route is found. This change in cross-linking is related to irreversible accumulation of collagen in fibrotic tissues. Collagen containing hydroxyallysine-derived cross-links is more difficult to degrade than collagen containing allysine-derived cross-links. Inhibition of the formation of hydroxyallysine-derived cross-links in fibrosis is therefore likely to result in the formation of collagen that is easier to degrade, thereby preventing unwanted collagen accumulation.

In the present study, two genes involved in fibrotic processes, i.e. *Pcolce* and *Plod2*, were found down-regulated in the PFJ group. The procollagen C-endopeptidase enhancer 1 (*Pcolce*) gene encodes a glycoprotein which binds and drives the enzymatic cleavage of type I procollagen and heightens C-proteinase activity, hence increasing fibrotic processes [108]. The increase in hydroxyallysine-derived cross-links in fibrosis is the result of an overhydroxylation of lysine residues within the collagen telopeptides, a function carried out by the enzyme encoded by procollagen-lysine, 2-oxoglutarate 5-dioxygenase 2 (*Plod2*). *Plod2* is thus involved in fibrotic processes as well [120].

### PFJ up-regulated hepatic apolipoprotein genes, especially apolipoprotein A1

Metabolic pathways for the utilisation of carbohydrates and fats are intricately intertwined. In addition to having profound effects on carbohydrate metabolism, insulin also has important effects on lipid metabolism. One of these is to promote the synthesis of fatty acids in the liver when the organ is saturated with glycogen, and these fatty acids are then exported from the liver as lipoproteins, which are further catabolised in the circulation, eventually yielding free fatty acids for use by other tissues. Insulin resistance and T2DM are associated with plasma lipid and lipoprotein abnormalities, which include reduced high-density lipoproteins (HDL), a predominance of low-density lipoproteins (LDL) and elevated TG levels, also previously described in NRs with T2DM [14]. Increased hepatic secretion of very-low-density lipoproteins (VLDL) and their impaired clearance also appear to be of central importance in the pathophysiology of this diabetic dyslipaemia [62]. In T2DM, increased efflux of free fatty acids from adipose tissues and impaired insulin-mediated skeletal muscle uptake of free fatty acids also increase fatty acid flux to the liver [11, 59]. Epidemiologic studies have demonstrated a relationship between insulin resistance and plasma free fatty acid levels [93]. In line with this, agents that lower elevated free fatty acids, such as thiazolidinediones, have been shown to improve insulin sensitivity in muscle, liver and adipose tissues [76, 78].

In the present study, genes up-regulated in the livers of NRs given PFJ include those encoding for apolipoproteins. The up-regulation of apolipoprotein genes, including *Apoa1*, *Apoa2*, *Apoc1* and *Apoc3*, suggests an increase in HDL synthesis relative to controls, as all apolipoproteins A1, A2, C1 and C3 are components of HDL. The first step in HDL synthesis involves the secretion of apolipoprotein A1 mainly by the liver and the intestine [132, 133]. Apolipoproteins A1 and A2 are the main scaffold proteins that determine HDL particle structure [13]. Apolipoprotein A1 levels are reported to be inversely associated with diabetic retinopathy [51]. Apolipoproteins C are constituents of chylomicrons, VLDL and HDL [55]. However, in the fasting state, apolipoproteins C are mainly associated with HDL, whereas in the fed state, they preferentially redistribute to the surface of chylomicrons and VLDL [73]. Apolipoprotein C1 overexpression in transgenic mice has been associated with protection from obesity and insulin resistance [56]. On the contrary, apolipoprotein C3 deficiency has been reported to result in diet-induced obesity and aggravated insulin resistance in mice [31].

Virtually, every lipid and lipoprotein is affected by insulin resistance and T2DM, but the control of hyperglycaemia is unlikely to correct existing dyslipaemia. Although plasma glucose control is important in reducing microvascular complications due to T2DM, lipid management is also essential in these patients to decrease the incidence of cardiovascular events. In the present study, the up-regulation of apolipoproteins important in HDL synthesis appeared beneficial, as evidenced by the significantly lower amounts of plasma TG (*p* < 0.05) and adipose tissues (*p* < 0.05) in NRs given PFJ compared to the control group. Although we did not measure the levels of HDL in the present study, we have previously shown that PFJ increased plasma HDL levels of golden Syrian hamsters fed an atherogenic diet [6]. In line with this, green tea extract rich in phenolic compounds was also previously found to significantly reduce fasting TG and increase HDL in within-group analysis of people with T2DM, in addition to causing a decreasing trend of fasting TG in between-group analysis [69]. The increase in apolipoprotein A1 in these T2DM patients is also comparable with that in HDL after green tea extract supplementation [69].

### Phase I and phase II detoxification genes were up-regulated in the livers of NRs given PFJ

Phase I and phase II detoxification enzyme systems are involved in the degradation of xenobiotics. To some extent, phenolic compounds in general may be regarded as xenobiotics by animal cells and are treated as such through interactions with these enzymes [81]. Phase I detoxification in the liver involves the activation of a series of enzymes called the cytochrome P450 mixed-function oxidases. These biotransformation enzymes function by oxidising, reducing or hydrolysing xenobiotics thus creating biotransformed intermediates [90]. Several cytochrome P450 genes involved in phase I detoxification, such as *Cyp1a2*, *Cyp2c67*, *Cyp2e1* and *Cyp4f14*, were up-regulated in NRs given PFJ. This is consistent with our previous observations, whereby cytochrome P450 genes were also up-regulated in mice given PFJ [65]. Conversely, hepatic *Cyp1a2* was found down-regulated in diabetic and insulin resistant New Zealand obese mice [89], while a decrease in hepatic *Cyp2e1* activity was reported in ob/ob mice and fa/fa Zucker rats [34]. *Cyp4f14* plays a role in the inactivation of eicosanoids [60], which could be beneficial in reducing inflammation.

Phase II detoxification enzymes perform conjugation reactions such as acylation, acetylation, glucuronidation, methylation, sulfation and glutathione conjugation, which help to convert biotransformed intermediates into less toxic, water-soluble substances that are easily excreted or eliminated from the body [90]. Incidentally, three antioxidant genes involved in phase II detoxification, i.e. *Ugt2b36*, *Cat* and *Gsto2*, were up-regulated in the livers of NRs given PFJ. *Ugt2b36* (uridine diphosphate glucuronosyltransferase 2 family, polypeptide B36) is a glycosyltransferase enzyme that catalyses the transfer of the glucuronic acid component of uridine diphosphate glucuronic acid to xenobiotics. *Ugt2b36* messenger ribonucleic acid (mRNA) levels were found to decrease in aging mice [39]. *Cat* (catalase) is a very important enzyme which protects cells from oxidative damage, as it catalyses the decomposition of hydrogen peroxide to water and oxygen. Blood catalase activity in T2DM subjects was found decreased when compared to that in non-diabetic controls, and this consequently increased hydrogen peroxide in muscle cells [43]. *Gsto2* (glutathione S-transferase omega-2) is an enzyme involved in glutathione conjugation. Patients with uncontrolled T2DM have severely deficient synthesis of glutathione attributed to limited precursor availability [104]. In addition, insulin administration is known to increase glutathione S-transferase gene expression through the PI3K/AKT/mTOR pathway and decrease intracellular oxidative stress [36].

### Real-time qRT-PCR validated the microarray data obtained

In the present study, the directions of fold changes of the target genes obtained from the real-time qRT-PCR technique as quantified by the qBase software [48] were comparable to those obtained from the microarray technique (Fig. 3). However, the magnitudes of fold changes obtained using real-time qRT-PCR were consistently lower than those obtained using microarrays. This has been described as the fold change compression phenomenon, which is caused by various technical microarray limitations, including limited dynamic range, signal saturations and cross hybridisations [127].

### Anti-diabetic effects of polyphenols and glucose homeostasis: does PFJ affect glucose absorption, insulin secretion or insulin sensitivity?

In addition to improving insulin production and function, another approach to overcome T2DM is to reduce glucose absorption by inhibiting the activities of digestive enzymes for glucose release/production or those of enterocyte membrane transporters responsible for glucose transport. Phenolic compounds have been reported to influence the apparent glycaemic indices of foods and limit postprandial glucose increases through these mechanisms [129]. For instance, phenolic compounds from certain fruits have been shown to inhibit activities of α-amylase and α-glucosidase [77], and some even have the potential to replace or reduce the dose of acarbose required during clinical trials to improve postprandial glycaemic control in T2DM [10]. Enterocyte membrane transporters responsible for glucose absorption in the small intestine include sodium-dependent glucose transporter 1 (SGLT1) and glucose transporter 2 (GLUT2). SGLT1 is responsible for glucose entrance from the apical side of the intestinal lumen into enterocytes via active transport, while GLUT2 assists glucose exit from the basolateral side of the intestinal lumen into the hepatic portal vein via facilitated diffusion [102]. Phenolic compounds have also been shown to inhibit these two types of transporters in human intestinal Caco-2 cell lines [54, 74].

We previously suggested that PFJ may slow the rate of glucose absorption, enhance insulin secretion and/or increase insulin sensitivity [16]. The results obtained in the present study indicate that the anti-diabetic effects of PFJ are likely due to mechanisms other than an increase in insulin secretion. This is because plasma insulin was not increased after PFJ supplementation in NRs, and another previous study also revealed that the early problem in NRs was insulin resistance with hyperinsulinaemia, not insulin insufficiency [15]. Nonetheless, it would be useful to conduct an insulin tolerance test on these NRs to further differentiate these two possible mechanisms.

### Insulin signalling in relation to longevity and chronic diseases: could the positive health effects of PFJ be attributed to modulation of insulin signalling?

The insulin-signalling pathway is an evolutionarily conserved mechanism of longevity from yeast to humans [7]. Therefore, modulation of this pathway has been suggested as an avenue in extending longevity and battling chronic diseases. Ample genetic evidence demonstrates that mild inhibition of insulin-signalling components (including the insulin receptor, IRS proteins and PI3Ks) or overactivation of forkhead box protein O (FoxO) transcription factors contributes to lifespan extension with improved metabolic profiles [49, 113]. Interestingly, Ayyadevara et al. [3] reported that genetic disruption of insulin-like signalling extended lifespan in the nematode *Caenorhabditis elegans* and to a lesser degree in other taxa including fruit flies and mice. They found remarkable longevity and stress resistance of nematode PI3K-null mutants that lacked the PI3K catalytic subunit [3]. Interestingly, the PI3K pathway has paradoxically two opposite functions, i.e. impairment of its signalling activates FoxO factors and extends lifespan, whereas its overactivity triggers nuclear factor-kappa beta (NF-κβ) signalling and accelerates the aging process. FoxO activation also causes concomitant enhancement of cellular stress resistance and protection, suppression of low-grade inflammation and enhanced mitochondrial biogenesis [121]. NF-κβ signalling has been recognised as one of the targets of PI3K pathway. The NF-κβ system is a pleiotropic factor regulating developmental processes, host defence systems and cellular survival functions [97]. Since the suppression of PI3K signalling can extend lifespan, this implies that excessive and sustained activation of PI3K signalling triggers the aging process.

In addition, there is increasing evidence for an association between obesity, T2DM and cancer. Epidemiologic data suggest that insulin resistance with hyperinsulinaemia, as well as increased insulin and insulin-like growth factor-1 (IGF-1) signalling account for the relationship between these conditions. Besides influencing T2DM, the PI3K pathway itself is also implicated in cancer. PI3K signalling is activated in human cancers via several different mechanisms, including direct mutational activation or amplification of genes encoding key components of the PI3K pathway. Activation of the PI3K pathway results in the activation of protein kinase B or AKT. AKT inhibits apoptosis and stimulates protein synthesis and cell proliferation. The fact that insulin receptor signalling can stimulate protein synthesis and inhibit apoptosis and the fact that IGF-1 receptor signalling enhances cell proliferation explain how hyperinsulinaemia and increased IGF-1 may result in tumour growth. These pathways thus represent an intricate balance, and disruption of this equilibrium may lead to obesity, T2DM and cancer. Uncontrolled signalling through the PI3K pathway also contributes to metastatic cancers [72]. Thus, understanding the intricacies of the PI3K pathway may provide new avenues in terms of extending longevity and overcoming chronic diseases [20].

It is thus exciting to find that PFJ down-regulated insulin signalling in the present study, as this pathway is a potential target for modulation of longevity and chronic diseases. It is also important to note that the *Pik3r3* gene, down-regulated in the livers of NRs given PFJ in the present study, is considered an oncogene important for cell proliferation and tumour growth, as it is overexpressed in certain cancers [126]. It is also interesting, but not surprising, that the gene expression patterns with regards to insulin signalling observed in the present study were not found in previous hepatic transcriptomic analyses of BALB/c mice tested on a low-fat diet [65] (with the exception of up-regulated cytochrome P450 genes), given a high-fat atherogenic diet [67] or injected with myeloma cells [66], as mice are not predisposed to T2DM since they are HDL animals in general and do not easily develop the metabolic syndrome. Nevertheless, we have previously shown that PFJ displayed many beneficial effects on degenerative diseases in various animal models [65–68, 99, 100, 103]. Therefore, from the results obtained in the present study, it would be noteworthy in future studies to investigate whether PFJ confers its positive effects on these diseases by modulating components of the insulin-signalling pathway, especially PI3Ks.

### Limitations of study

We acknowledge that a limitation in the present study was that mouse (*Mus musculus*) microarrays and real-time qRT-PCR assays were used to assess the gene expression changes of the NR (*Arvicanthis niloticus*). However, the application of the NR as a laboratory diurnal rodent for biomedical research applicable to humans is relatively new [94]. Therefore, detailed knowledge of its physiology is still lacking, and its genome has not been sequenced. Accordingly, no commercial whole genome microarrays are currently available for this species. Nevertheless, cross hybridisation studies using microarrays have been conducted previously, such as studies involving hybridising monkey samples to human microarrays [25, 29, 42, 52, 63, 75]. NRs belong to the Muridae family, as do mice and rats [124]. As with the standard laboratory rat, the NR is relatively insensitive to variations in photoperiod and does not hibernate. Compared to the standard laboratory rat however, the NR reaches asymptotic body mass early in life and does not show marked sexual dimorphism [94]. We have previously tried hybridising NR samples to rat (*Rattus norvegicus*) microarrays, but quality control of the hybridisation indicated that the hybridisation was not satisfactory (Vassilis Zannis, Boston University School of Medicine, MA, personal communication). On the other hand, the hybridisation of NR samples to mouse (*Mus musculus*) microarrays carried out in the present study was of high quality, enabling interpretation of the data obtained. Nevertheless, future studies to delve further into the transcriptomic effects of PFJ on NRs would benefit from the various next-generation sequencing technologies and platforms currently available. It would also be interesting to compare the effects of PFJ in different animal models, especially to identify whether species-specific genes are involved.

Another limitation in the present study was that microarray gene expression profiling was not carried out on pancreatic islet β cells, the site for insulin production. Obtaining high-quality and intact RNA from the pancreatic β cells is difficult, however, as the primary function of the pancreas is as an exocrine aid in digestion. The pancreas thus expresses large quantities of proteases, DNases and RNases that initiate an autolytic process almost immediately upon harvest [83]. In addition, some techniques also involve tedious pancreatic cannulation procedures and cause tissue artefacts. However, newer and simpler techniques are emerging, such as the perfusion method using RNase inhibitors [45] and modifications of standard phenol/guanidine thiocyanate lysis reagent protocols [4]. These emerging protocols could be used in future experiments to study the gene expression changes caused by PFJ in the pancreas.

## Conclusions

Transcriptomic gene expression analysis using microarrays from the livers of young male NRs supplemented with PFJ to prevent T2DM induction showed that genes related to HDL apolipoproteins and hepatic detoxification were up-regulated, while genes related to insulin signalling and fibrosis were down-regulated. Based on the results obtained, it is more likely that the anti-diabetic effects of PFJ may be due to mechanisms other than an increase in insulin secretion, as the levels of insulin were not increased after PFJ supplementation in NRs, and young NRs have high concentrations of insulin during diabetes induction that suggest insulin resistance is the primary defect [15]. Further studies to investigate whether PFJ confers its positive effects on degenerative diseases by modulating components of the insulin-signalling pathway are also warranted.

## Abbreviations

ANOVA: Analysis of variance
cDNA: Complementary deoxyribonucleic acid
cRNA: Complementary ribonucleic acid
Ct: Threshold cycle
En: Energy
FBG: Fasting blood glucose
FoxO: Forkhead box protein O
GAE: Gallic acid equivalent
GLUT2: Glucose transporter 2
GLUT4: Glucose transporter 4
HDL: High-density lipoproteins
IGF-1: Insulin-like growth factor 1
IRS: Insulin receptor substrate
LDL: Low-density lipoproteins
MAPK: Mitogen-activated protein kinase
mRNA: Messenger ribonucleic acid
mTOR: Mammalian target of rapamycin
NF-κβ: Nuclear factor-kappa beta
NR: Nile rat
NTC: Non-template control
PFJ: Palm fruit juice
PI3K: Phosphatidylinositol 3-kinase
qRT-PCR: Quantitative reverse transcription-polymerase chain reaction
RBG: Random blood glucose
SD: Standard deviation
SGLT1: Sodium-dependent glucose transporter 1
T2DM: Type 2 diabetes mellitus
TC: Total cholesterol
TG: Triacylglycerol
VLDL: Very-low-density lipoproteins

## References

[1] Anhe FF, Desjardins Y, Pilon G, Dudonne S, Genovese MI, Lajolo FM, Marette A (2013). Polyphenols and type 2 diabetes: a prospective review. Pharma Nutr.

[2] Aprikian O, Duclos V, Guyot S, Besson C, Manach C, Bernalier A, Morand C, Remesy C, Demigne C (2003). Apple pectin and a polyphenol-rich apple concentrate are more effective together than separately on cecal fermentations and plasma lipids in rats. J Nutr.

[3] Ayyadevara S, Alla R, Thaden JJ, Shmookler Reis RJ (2008). Remarkable longevity and stress resistance of nematode PI3K-null mutants. Aging Cell.

[4] Azevedo-Pouly AC, Elgamal OA, Schmittgen TD (2014). RNA isolation from mouse pancreas: a ribonuclease-rich tissue. J Vis Exp.

[5] Bahadoran Z, Mirmiran P, Azizi F (2013). Dietary polyphenols as potential nutraceuticals in management of diabetes: a review. J Diabetes Metab Disord.

[6] Balasundram N, Sundram K, Samman S (2005). Phenolic-rich palm fruit juice raises plasma HDL-C concentrations and improves antioxidant status in Golden Syrian hamsters fed an atherogenic diet. Asia Pac J Clin Nutr.

[7] Barbieri M, Bonafe M, Franceschi C, Paolisso G (2003). Insulin/IGF-I-signaling pathway: an evolutionarily conserved mechanism of longevity from yeast to humans. Am J Physiol Endocrinol Metab.

[8] Bauer F, Beulens JW, Van der AD, Wijmenga C, Grobbee DE, Spijkerman AM, Van der Schouw YT, Onland-Moret NC (2013). Dietary patterns and the risk of type 2 diabetes in overweight and obese individuals. Eur J Nutr.

[9] Bhupathiraju SN, Tobias DK, Malik VS, Pan A, Hruby A, Manson JE, Willett WC, Hu FB (2014). Glycemic index, glycemic load, and risk of type 2 diabetes: results from 3 large US cohorts and an updated meta-analysis. Am J Clin Nutr.

[10] Boath AS, Stewart D, McDougall GJ (2012). Berry components inhibit alpha-glucosidase in vitro: synergies between acarbose and polyphenols from black currant and rowanberry. Food Chem.

[11] Boden G (1997). Role of fatty acids in the pathogenesis of insulin resistance and NIDDM. Diabetes.

[12] Bogan JS (2012). Regulation of glucose transporter translocation in health and diabetes. Annu Rev Biochem.

[13] Bolanos-Garcia VM, Miguel RN (2003). On the structure and function of apolipoproteins: more than a family of lipid-binding proteins. Prog Biophys Mol Biol.

[14] Bolsinger J, Pronczuk A, Hayes KC (2013). Dietary carbohydrate dictates development of type 2 diabetes in the Nile rat. J Nutr Biochem.

[15] Bolsinger J, Pronczuk A, Landstrom M, Auerbach A, Hayes KC. Low glycemic load diets protect against metabolic syndrome and type 2 diabetes mellitus in the Nile rat. J Nutr Biochem. 2016, In press. 10.1016/j.jnutbio.2017.01.007 28187365

[16] Bolsinger J, Pronczuk A, Sambanthamurthi R, Hayes KC (2014). Anti-diabetic effects of palm fruit juice in the Nile rat (*Arvicanthis niloticus*). J Nutr Sci.

[17] Bray GA, Lovejoy JC, Smith SR, DeLany JP, Lefevre M, Hwang D, Ryan DH, York DA (2002). The influence of different fats and fatty acids on obesity, insulin resistance and inflammation. J Nutr.

[18] Bryant NJ, Govers R, James DE (2002). Regulated transport of the glucose transporter GLUT4. Nat Rev Mol Cell Biol.

[19] Bustin SA, Nolan T (2004). Pitfalls of quantitative real-time reverse-transcription polymerase chain reaction. J Biomol Tech.

[20] Cantley LC (2002). The phosphoinositide 3-kinase pathway. Science.

[21] Chaabo F, Pronczuk A, Maslova E, Hayes K (2010). Nutritional correlates and dynamics of diabetes in the Nile rat (*Arvicanthis niloticus*): a novel model for diet-induced type 2 diabetes and the metabolic syndrome. Nutr Metab.

[22] Che Idris CA, Karupaiah T, Sundram K, Tan YA, Balasundram N, Leow SS, Nasruddin NS, Sambanthamurthi R (2014). Oil palm phenolics and vitamin E reduce atherosclerosis in rabbits. J Funct Foods.

[23] Chen D, Mauvais-Jarvis F, Bluher M, Fisher SJ, Jozsi A, Goodyear LJ, Ueki K, Kahn CR (2004). p50alpha/p55alpha phosphoinositide 3-kinase knockout mice exhibit enhanced insulin sensitivity. Mol Cell Biol.

[24] Chiang DJ, Pritchard MT, Nagy LE (2011). Obesity, diabetes mellitus, and liver fibrosis. Am J Physiol Gastrointest Liver Physiol.

[25] Chismar JD, Mondala T, Fox HS, Roberts E, Langford D, Masliah E, Salomon DR, Head SR (2002). Analysis of result variability from high-density oligonucleotide arrays comparing same-species and cross-species hybridizations. Biotechniques.

[26] Cuervo A, Valdes L, Salazar N, De los Reyes-Gavilan CG, Ruas-Madiedo P, Gueimonde M, Gonzalez S (2014). Pilot study of diet and microbiota: interactive associations of fibers and polyphenols with human intestinal bacteria. J Agric Food Chem.

[27] Czech MP, Corvera S (1999). Signaling mechanisms that regulate glucose transport. J Biol Chem.

[28] De Bock M, Derraik JG, Brennan CM, Biggs JB, Morgan PE, Hodgkinson SC, Hofman PL, Cutfield WS (2013). Olive (*Olea europaea* L.) leaf polyphenols improve insulin sensitivity in middle-aged overweight men: a randomized, placebo-controlled, crossover trial. PLoS One.

[29] Dillman JF, Phillips CS (2005). Comparison of non-human primate and human whole blood tissue gene expression profiles. Toxicol Sci.

[30] Duda-Chodak A, Tarko T, Satora P, Sroka P (2015). Interaction of dietary compounds, especially polyphenols, with the intestinal microbiota: a review. Eur J Nutr.

[31] Duivenvoorden I, Teusink B, Rensen PC, Romijn JA, Havekes LM, Voshol PJ (2005). Apolipoprotein C3 deficiency results in diet-induced obesity and aggravated insulin resistance in mice. Diabetes.

[32] Eckel RH, Alberti KG, Grundy SM, Zimmet PZ (2010). The metabolic syndrome. Lancet.

[33] Edgar R, Domrachev M, Lash AE (2002). Gene Expression Omnibus: NCBI gene expression and hybridization array data repository. Nucleic Acids Res.

[34] Enriquez A, Leclercq I, Farrell GC, Robertson G (1999). Altered expression of hepatic CYP2E1 and CYP4A in obese, diabetic ob/ob mice, and fa/fa Zucker rats. Biochem Biophys Res Commun.

[35] Eshak ES, Iso H, Mizoue T, Inoue M, Noda M, Tsugane S (2013). Soft drink, 100 % fruit juice, and vegetable juice intakes and risk of diabetes mellitus. Clin Nutr.

[36] Franco R, Schoneveld OJ, Pappa A, Panayiotidis MI (2007). The central role of glutathione in the pathophysiology of human diseases. Arch Physiol Biochem.

[37] Frejnagel S, Juskiewicz J (2011). Dose-dependent effects of polyphenolic extracts from green tea, blue-berried honeysuckle, and chokeberry on rat caecal fermentation processes. Planta Med.

[38] Fruman DA, Mauvais-Jarvis F, Pollard DA, Yballe CM, Brazil D, Bronson RT, Kahn CR, Cantley LC (2000). Hypoglycaemia, liver necrosis and perinatal death in mice lacking all isoforms of phosphoinositide 3-kinase p85 alpha. Nat Genet.

[39] Fu ZD, Csanaky IL, Klaassen CD (2012). Effects of aging on mRNA profiles for drug-metabolizing enzymes and transporters in livers of male and female mice. Drug Metab Dispos.

[40] Geering B, Cutillas PR, Vanhaesebroeck B (2007). Regulation of class IA PI3Ks: is there a role for monomeric PI3K subunits?. Biochem Soc Trans.

[41] Gehart H, Kumpf S, Ittner A, Ricci R (2010). MAPK signalling in cellular metabolism: stress or wellness?. EMBO Rep.

[42] George MD, Sankaran S, Reay E, Gelli AC, Dandekar S (2003). High-throughput gene expression profiling indicates dysregulation of intestinal cell cycle mediators and growth factors during primary simian immunodeficiency virus infection. Virology.

[43] Goth L (2008). Catalase deficiency and type 2 diabetes. Diabetes Care.

[44] Graham TE, Kahn BB (2007). Tissue-specific alterations of glucose transport and molecular mechanisms of intertissue communication in obesity and type 2 diabetes. Horm Metab Res.

[45] Griffin M, Abu-El-Haija M, Abu-El-Haija M, Rokhlina T, Uc A (2012). Simplified and versatile method for isolation of high-quality RNA from pancreas. Biotechniques.

[46] Groop L, Pociot F (2014). Genetics of diabetes—are we missing the genes or the disease?. Mol Cell Endocrinol.

[47] Hanhineva K, Torronen R, Bondia-Pons I, Pekkinen J, Kolehmainen M, Mykkanen H, Poutanen K (2010). Impact of dietary polyphenols on carbohydrate metabolism. Int J Mol Sci.

[48] Hellemans J, Mortier G, De Paepe A, Speleman F, Vandesompele J (2007). qBase relative quantification framework and software for management and automated analysis of real-time quantitative PCR data. Genome Biol.

[49] Holzenberger M, Kappeler L, De Magalhaes FC (2004). IGF-1 signaling and aging. Exp Gerontol.

[50] Huang PL (2009). A comprehensive definition for metabolic syndrome. Dis Model Mech.

[51] Irshad M, Dubey R (2005). Apolipoproteins and their role in different clinical conditions: an overview. Indian J Biochem Biophys.

[52] Jacquelin B, Mayau V, Brysbaert G, Regnault B, Diop OM, Arenzana-Seisdedos F, Rogge L, Coppee JY, Barre-Sinoussi F, Benecke A, Muller-Trutwin MC (2007). Long oligonucleotide microarrays for African green monkey gene expression profile analysis. FASEB J.

[53] Johnson IT (2002). Anticarcinogenic effects of diet-related apoptosis in the colorectal mucosa. Food Chem Toxicol.

[54] Johnston K, Sharp P, Clifford M, Morgan L (2005). Dietary polyphenols decrease glucose uptake by human intestinal Caco-2 cells. FEBS Lett.

[55] Jong MC, Hofker MH, Havekes LM (1999). Role of ApoCs in lipoprotein metabolism: functional differences between ApoC1, ApoC2, and ApoC3. Arterioscler Thromb Vasc Biol.

[56] Jong MC, Voshol PJ, Muurling M, Dahlmans VE, Romijn JA, Pijl H, Havekes LM (2001). Protection from obesity and insulin resistance in mice overexpressing human apolipoprotein C1. Diabetes.

[57] Kaur J (2014). A comprehensive review on metabolic syndrome. Cardiol Res Pract.

[58] Kelder T, van Iersel MP, Hanspers K, Kutmon M, Conklin BR, Evelo CT, Pico AR (2012). WikiPathways: building research communities on biological pathways. Nucleic Acids Res.

[59] Kelley DE, Simoneau JA (1994). Impaired free fatty acid utilization by skeletal muscle in non-insulin-dependent diabetes mellitus. J Clin Invest.

[60] Kikuta Y, Kasyu H, Kusunose E, Kusunose M (2000). Expression and catalytic activity of mouse leukotriene B4 omega-hydroxylase, CYP4F14. Arch Biochem Biophys.

[61] Kim HM, Kim J (2013). The effects of green tea on obesity and type 2 diabetes. Diabetes Metab J.

[62] Krauss RM (2004). Lipids and lipoproteins in patients with type 2 diabetes. Diabetes Care.

[63] Lachance PE, Chaudhuri A (2004). Microarray analysis of developmental plasticity in monkey primary visual cortex. J Neurochem.

[64] Lee C, Longo V (2016) Dietary restriction with and without caloric restriction for healthy aging. F1000Res 5. 10.12688/f1000research.7136.1 PMC4755412 26918181

[65] Leow SS, Sekaran SD, Sundram K, Tan YA, Sambanthamurthi R (2011). Differential transcriptomic profiles effected by oil palm phenolics indicate novel health outcomes. BMC Genomics.

[66] Leow SS, Sekaran SD, Sundram K, Tan YA, Sambanthamurthi R. Gene expression changes in spleens and livers of tumour-bearing mice suggest delayed inflammation and attenuated cachexia in response to oil palm phenolics. J Nutrigenet Nutrigenomics. 2013a;6(6):305-326. 10.1159/000357948 24642698

[67] Leow SS, Sekaran SD, Sundram K, Tan YA, Sambanthamurthi R. Oil palm phenolics attenuate changes caused by an atherogenic diet in mice. Eur J Nutr. 2013b;52(2):443-456. 10.1007/s00394-012-0346-0 PMC3573186 22527284

[68] Leow SS, Sekaran SD, Tan YA, Sundram K, Sambanthamurthi R. Oil palm phenolics confer neuroprotective effects involving cognitive and motor functions in mice. Nutr Neurosci. 2013c;16(5):207-217. 10.1179/1476830512Y.0000000047 PMC3736891 23433062

[69] Liu CY, Huang CJ, Huang LH, Chen IJ, Chiu JP, Hsu CH (2014). Effects of green tea extract on insulin resistance and glucagon-like peptide 1 in patients with type 2 diabetes and lipid abnormalities: a randomized, double-blinded, and placebo-controlled trial. PLoS One.

[70] Lyons J, Brown F, Remillard DE, Bolsinger J, Hayes KC. Pathology of the Nile rat developing type 2 diabetes [abstract]. Faseb J. 2013;27(Meeting Abstract Supplement):874.813.

[71] Lyssenko V, Jonsson A, Almgren P, Pulizzi N, Isomaa B, Tuomi T, Berglund G, Altshuler D, Nilsson P, Groop L (2008). Clinical risk factors, DNA variants, and the development of type 2 diabetes. N Engl J Med.

[72] Maehama T, Dixon JE (1999). PTEN: a tumour suppressor that functions as a phospholipid phosphatase. Trends Cell Biol.

[73] Mahley RW, Innerarity TL, Rall SC, Weisgraber KH (1984). Plasma lipoproteins: apolipoprotein structure and function. J Lipid Res.

[74] Manzano S, Williamson G (2010). Polyphenols and phenolic acids from strawberry and apple decrease glucose uptake and transport by human intestinal Caco-2 cells. Mol Nutr Food Res.

[75] Marvanova M, Menager J, Bezard E, Bontrop RE, Pradier L, Wong G (2003). Microarray analysis of nonhuman primates: validation of experimental models in neurological disorders. FASEB J.

[76] Mayerson AB, Hundal RS, Dufour S, Lebon V, Befroy D, Cline GW, Enocksson S, Inzucchi SE, Shulman GI, Petersen KF (2002). The effects of rosiglitazone on insulin sensitivity, lipolysis, and hepatic and skeletal muscle triglyceride content in patients with type 2 diabetes. Diabetes.

[77] McDougall GJ, Shpiro F, Dobson P, Smith P, Blake A, Stewart D (2005). Different polyphenolic components of soft fruits inhibit alpha-amylase and alpha-glucosidase. J Agric Food Chem.

[78] Miyazaki Y, Mahankali A, Matsuda M, Mahankali S, Hardies J, Cusi K, Mandarino LJ, DeFronzo RA (2002). Effect of pioglitazone on abdominal fat distribution and insulin sensitivity in type 2 diabetic patients. J Clin Endocrinol Metab.

[79] Moco S, Martin FP, Rezzi S (2012). Metabolomics view on gut microbiome modulation by polyphenol-rich foods. J Proteome Res.

[80] Morimoto A, Ohno Y, Tatsumi Y, Mizuno S, Watanabe S (2012). Effects of healthy dietary pattern and other lifestyle factors on incidence of diabetes in a rural Japanese population. Asia Pac J Clin Nutr.

[81] Moskaug JO, Carlsen H, Myhrstad MC, Blomhoff R (2005). Polyphenols and glutathione synthesis regulation. Am J Clin Nutr.

[82] Movahed A, Nabipour I, Lieben Louis X, Thandapilly SJ, Yu L, Kalantarhormozi M, Rekabpour SJ, Netticadan T (2013). Antihyperglycemic effects of short term resveratrol supplementation in type 2 diabetic patients. Evid Based Complement Alternat Med.

[83] Mullin AE, Soukatcheva G, Verchere CB, Chantler JK (2006). Application of in situ ductal perfusion to facilitate isolation of high-quality RNA from mouse pancreas. Biotechniques.

[84] Neyrinck AM, Van Hee VF, Bindels LB, De Backer F, Cani PD, Delzenne NM (2013). Polyphenol-rich extract of pomegranate peel alleviates tissue inflammation and hypercholesterolaemia in high-fat diet-induced obese mice: potential implication of the gut microbiota. Br J Nutr.

[85] Noda K, Melhorn MI, Zandi S, Frimmel S, Tayyari F, Hisatomi T, Almulki L, Pronczuk A, Hayes KC, Hafezi-Moghadam A (2010). An animal model of spontaneous metabolic syndrome: Nile grass rat. Faseb J.

[86] Nolan T, Hands RE, Bustin SA (2006). Quantification of mRNA using real-time RT-PCR. Nat Protoc.

[87] Olokoba AB, Obateru OA, Olokoba LB (2012). Type 2 diabetes mellitus: a review of current trends. Oman Med J.

[88] Osman HF, Eshak MG, El-Sherbiny EM, Bayoumi MM (2012). Biochemical and genetical evaluation of pomegranate impact on diabetes mellitus induced by alloxan in female rats. Life Sci J.

[89] Pass GJ, Becker W, Kluge R, Linnartz K, Plum L, Giesen K, Joost HG (2002). Effect of hyperinsulinemia and type 2 diabetes-like hyperglycemia on expression of hepatic cytochrome p450 and glutathione s-transferase isoforms in a New Zealand obese-derived mouse backcross population. J Pharmacol Exp Ther.

[90] Percival M (1997). Phytonutrients and detoxification. Clin Nutr Insights.

[91] Pessin JE, Saltiel AR (2000). Signaling pathways in insulin action: molecular targets of insulin resistance. J Clin Invest.

[92] Pico AR, Kelder T, van Iersel MP, Hanspers K, Conklin BR, Evelo C (2008). WikiPathways: pathway editing for the people. PLoS Biol.

[93] Reaven GM, Chen YD (1988). Role of abnormal free fatty acid metabolism in the development of non-insulin-dependent diabetes mellitus. Am J Med.

[94] Refinetti R (2004). The Nile grass rat as a laboratory animal. Lab Anim (NY).

[95] Romo-Vaquero M, Selma MV, Larrosa M, Obiol M, Garcia-Villalba R, Gonzalez-Barrio R, Issaly N, Flanagan J, Roller M, Tomas-Barberan FA, Garcia-Conesa MT (2014). A rosemary extract rich in carnosic acid selectively modulates caecum microbiota and inhibits beta-glucosidase activity, altering fiber and short chain fatty acids fecal excretion in lean and obese female rats. PLoS One.

[96] Sabio G, Davis RJ (2010). cJun NH2-terminal kinase 1 (JNK1): roles in metabolic regulation of insulin resistance. Trends Biochem Sci.

[97] Salminen A, Kaarniranta K (2010). Insulin/IGF-1 paradox of aging: regulation via AKT/IKK/NF-kappaB signaling. Cell Signal.

[98] Saltiel AR, Kahn CR (2001). Insulin signalling and the regulation of glucose and lipid metabolism. Nature.

[99] Sambanthamurthi R, Tan YA, Sundram K, Abeywardena M, Sambandan TG, Rha C, Sinskey AJ, Subramaniam K, Leow SS, Hayes KC, Wahid MB. Oil palm vegetation liquor: a new source of phenolic bioactives. Br J Nutr. 2011a;106(11):1655-1663. 10.1017/S0007114511002121 PMC4179495 21736792

[100] Sambanthamurthi R, Tan YA, Sundram K, Hayes KC, Abeywardena M, Leow SS, Sekaran SD, Sambandan TG, Rha C, Sinskey AJ, Subramaniam K, Fairus S, Wahid MB. Positive outcomes of oil palm phenolics on degenerative diseases in animal models. Br J Nutr. 2011b;106(11):1664-1675. 10.1017/S0007114511002133 PMC4179496 21736778

[101] Samuel VT, Shulman GI (2012). Mechanisms for insulin resistance: common threads and missing links. Cell.

[102] Scheepers A, Joost HG, Schurmann A (2004). The glucose transporter families SGLT and GLUT: molecular basis of normal and aberrant function. JPEN J Parenter Enteral Nutr.

[103] Sekaran SD, Leow SS, Abobaker N, Tee KK, Sundram K, Sambanthamurthi R, Wahid MB (2010). Effects of oil palm phenolics on tumor cells *in vitro* and *in vivo*. Afr J Food Sci.

[104] Sekhar RV, McKay SV, Patel SG, Guthikonda AP, Reddy VT, Balasubramanyam A, Jahoor F (2011). Glutathione synthesis is diminished in patients with uncontrolled diabetes and restored by dietary supplementation with cysteine and glycine. Diabetes Care.

[105] Shen J, Goyal A, Sperling L (2012). The emerging epidemic of obesity, diabetes, and the metabolic syndrome in china. Cardiol Res Pract.

[106] Shepherd PR, Withers DJ, Siddle K (1998). Phosphoinositide 3-kinase: the key switch mechanism in insulin signalling. Biochem J.

[107] Slavin J (2013). Fiber and prebiotics: mechanisms and health benefits. Nutrients.

[108] Steiglitz BM, Kreider JM, Frankenburg EP, Pappano WN, Hoffman GG, Meganck JA, Liang X, Hook M, Birk DE, Goldstein SA, Greenspan DS (2006). Procollagen C proteinase enhancer 1 genes are important determinants of the mechanical properties and geometry of bone and the ultrastructure of connective tissues. Mol Cell Biol.

[109] Stockli J, Fazakerley DJ, James DE (2011). GLUT4 exocytosis. J Cell Sci.

[110] Szkudelski T, Szkudelska K (2011). Anti-diabetic effects of resveratrol. Ann N Y Acad Sci.

[111] Taniguchi CM, Emanuelli B, Kahn CR. Critical nodes in signalling pathways: insights into insulin action. Nat Rev Mol Cell Biol. 2006a;7(2):85-96. 10.1038/nrm1837 16493415

[112] Taniguchi CM, Kondo T, Sajan M, Luo J, Bronson R, Asano T, Farese R, Cantley LC, Kahn CR. Divergent regulation of hepatic glucose and lipid metabolism by phosphoinositide 3-kinase via Akt and PKClambda/zeta. Cell Metab. 2006b;3(5):343-353. 10.1016/j.cmet.2006.04.005 16679292

[113] Tatar M, Bartke A, Antebi A (2003). The endocrine regulation of aging by insulin-like signals. Science.

[114] Terauchi Y, Tsuji Y, Satoh S, Minoura H, Murakami K, Okuno A, Inukai K, Asano T, Kaburagi Y, Ueki K, Nakajima H, Hanafusa T, Matsuzawa Y, Sekihara H, Yin Y, Barrett JC, Oda H, Ishikawa T, Akanuma Y, Komuro I, Suzuki M, Yamamura K, Kodama T, Suzuki H, Yamamura K, Kodama T, Suzuki H, Koyasu S, Aizawa S, Tobe K, Fukui Y, Yazaki Y, Kadowaki T (1999). Increased insulin sensitivity and hypoglycaemia in mice lacking the p85 alpha subunit of phosphoinositide 3-kinase. Nat Genet.

[115] Tolman KG, Fonseca V, Dalpiaz A, Tan MH (2007). Spectrum of liver disease in type 2 diabetes and management of patients with diabetes and liver disease. Diabetes Care.

[116] Tozzo E, Gnudi L, Kahn BB (1997). Amelioration of insulin resistance in streptozotocin diabetic mice by transgenic overexpression of GLUT4 driven by an adipose-specific promoter. Endocrinology.

[117] Trombetta M, Spiazzi G, Zoppini G, Muggeo M (2005). Review article: type 2 diabetes and chronic liver disease in the Verona diabetes study. Aliment Pharmacol Ther.

[118] Ueki K, Algenstaedt P, Mauvais-Jarvis F, Kahn CR (2000). Positive and negative regulation of phosphoinositide 3-kinase-dependent signaling pathways by three different gene products of the p85alpha regulatory subunit. Mol Cell Biol.

[119] Ueki K, Fruman DA, Yballe CM, Fasshauer M, Klein J, Asano T, Cantley LC, Kahn CR (2003). Positive and negative roles of p85 alpha and p85 beta regulatory subunits of phosphoinositide 3-kinase in insulin signaling. J Biol Chem.

[120] van der Slot AJ, Zuurmond AM, Bardoel AF, Wijmenga C, Pruijs HE, Sillence DO, Brinckmann J, Abraham DJ, Black CM, Verzijl N, DeGroot J, Hanemaaijer R, TeKoppele JM, Huizinga TW, Bank RA (2003). Identification of PLOD2 as telopeptide lysyl hydroxylase, an important enzyme in fibrosis. J Biol Chem.

[121] van Heemst D (2010). Insulin, IGF-1 and longevity. Aging Dis.

[122] van Iersel MP, Kelder T, Pico AR, Hanspers K, Coort S, Conklin BR, Evelo C (2008). Presenting and exploring biological pathways with PathVisio. BMC Bioinformatics.

[123] Vandesompele J, De Preter K, Pattyn F, Poppe B, Van Roy N, De Paepe A, Speleman F. Accurate normalization of real-time quantitative RT-PCR data by geometric averaging of multiple internal control genes. Genome Biol. 2002;3(7):RESEARCH0034. 10.1186/gb-2002-3-7-research0034 PMC126239 12184808

[124] Volobouev VT, Ducroz JF, Aniskin VM, Britton-Davidian J, Castiglia R, Dobigny G, Granjon L, Lombard M, Corti M, Sicard B, Capanna E (2002). Chromosomal characterization of Arvicanthis species (Rodentia, Murinae) from western and central Africa: implications for taxonomy. Cytogenet Genome Res.

[125] Wainstein J, Ganz T, Boaz M, Bar Dayan Y, Dolev E, Kerem Z, Madar Z (2012). Olive leaf extract as a hypoglycemic agent in both human diabetic subjects and in rats. J Med Food.

[126] Wang G, Yang X, Li C, Cao X, Luo X, Hu J (2014). PIK3R3 induces epithelial-to-mesenchymal transition and promotes metastasis in colorectal cancer. Mol Cancer Ther.

[127] Wang Y, Barbacioru C, Hyland F, Xiao W, Hunkapiller KL, Blake J, Chan F, Gonzalez C, Zhang L, Samaha RR (2006). Large scale real-time PCR validation on gene expression measurements from two commercial long-oligonucleotide microarrays. BMC Genomics.

[128] Wang YW, Sun GD, Sun J, Liu SJ, Wang J, Xu XH, Miao LN (2013). Spontaneous type 2 diabetic rodent models. J Diab Res.

[129] Williamson G (2013). Possible effects of dietary polyphenols on sugar absorption and digestion. Mol Nutr Food Res.

[130] Wu GD, Chen J, Hoffmann C, Bittinger K, Chen YY, Keilbaugh SA, Bewtra M, Knights D, Walters WA, Knight R, Sinha R, Gilroy E, Gupta K, Baldassano R, Nessel L, Li H, Bushman FD, Lewis JD (2011). Linking long-term dietary patterns with gut microbial enterotypes. Science.

[131] Yu J, Zhang Y, McIlroy J, Rordorf-Nikolic T, Orr GA, Backer JM (1998). Regulation of the p85/p110 phosphatidylinositol 3'-kinase: stabilization and inhibition of the p110alpha catalytic subunit by the p85 regulatory subunit. Mol Cell Biol.

[132] Zannis VI, Cole FS, Jackson CL, Kurnit DM, Karathanasis SK (1985). Distribution of apolipoprotein A-I, C-II, C-III, and E mRNA in fetal human tissues. Time-dependent induction of apolipoprotein E mRNA by cultures of human monocyte-macrophages. Biochemistry.

[133] Zannis VI, Fotakis P, Koukos G, Kardassis D, Ehnholm C, Jauhiainen M, Chroni A (2015). HDL biogenesis, remodeling, and catabolism. Handb Exp Pharmacol.

